# Use of Telemedicine in Pediatric Services for 4 Representative Clinical Conditions: Scoping Review

**DOI:** 10.2196/38267

**Published:** 2022-10-26

**Authors:** Genevieve Southgate, Arrash A Yassaee, Matthew J Harmer, Helen Livesey, Kate Pryde, Damian Roland

**Affiliations:** 1 Southampton Children's Hospital Southampton United Kingdom; 2 Centre for Paediatrics and Child Health Faculty of Medicine Imperial College London United Kingdom; 3 Huma Therapeutics London United Kingdom; 4 Leeds Teaching Hospitals National Health Service Trust Leeds United Kingdom; 5 Social Science Applied Healthcare and Improvement Research (SAPPHIRE) Group Health Sciences University of Leicester National Health Service Trust Leicester United Kingdom; 6 Paediatric Emergency Medicine Leicester Academic (PEMLA) Group University Hospitals of Leicester National Health Service Trust Leicester United Kingdom

**Keywords:** telemedicine, telehealth, eHealth, digital health, video consultation, remote consultation, paediatric, child, safeguarding, diabetes, diabetic, asthma, epilepsy, epileptic, renal, kidney, evidence-based medicine, review

## Abstract

**Background:**

Telemedicine is becoming routine in health care. Postpandemic, a universal return to face-to-face consultations may risk a loss of some of the advantages of telemedicine. However, rapid implementation and adoption without robust evaluation of usability, efficacy, and effectiveness could potentially lead to suboptimal health outcomes and downstream challenges to providers.

**Objective:**

This review assesses telemedicine interventions against international guidance and sufficiency of evidence to support postpandemic utilization in pediatric settings.

**Methods:**

This scoping review was performed following searches on PubMed, Embase, and CINAHL databases on April 15, 2021, and May 31, 2022, and examined studies focused on telemedicine, remote consultation, video call, or remote patient monitoring in children (0-18 years) receiving outpatient care for diabetes, asthma, epilepsy, or renal disease. Exclusion criteria included studies published before 2011 as the technologies used have likely been improved or replaced, studies in adult populations or where it was not possible to disaggregate data for participants younger than 18 years as the focus of the review was on pediatric care, and studies not published in English. Data were extracted by 4 authors, and the data were corroborated by a second reviewer. Studies were examined for feasibility and usability, clinical and process outcomes, and cost-effectiveness.

**Results:**

Of the 3158 studies identified, 56 were suitable for final inclusion and analysis. Data on feasibility or usability of interventions (48 studies) were overwhelmingly positive in support of telemedicine interventions, with common themes including convenience, perceived cost savings, and ease of use. However, use in preference to usual care was rarely explored. Clinical and process outcome data (31 studies) were mostly positive. Across all studies, there was limited measurement of standardized clinical outcomes, although these were more commonly reported in asthma (peak flow) and diabetes (glycated hemoglobin [HbA1c]). Implementation science data generally supported cost-effectiveness of telemedicine with a reduction of health care costs.

**Conclusions:**

There is promising evidence supporting telemedicine in pediatric settings. However, there is a lack of evaluation of telemedicine in comparison with usual outpatient care for noninferiority of clinical outcomes, and this review highlights the need for a more standardized approach to evaluation of digital interventions.

## Introduction

Telemedicine is the practice of medicine using technology to provide remote health assessment and therapeutic intervention to a patient at a distant site. The spectrum is broad, from simple telephone and video consultations, through wearable digital monitoring, to complex experimental interventions with surgeons guiding robotic instruments to deliver remote surgery.

The adoption of telemedicine consultations escalated rapidly in response to the COVID-19 pandemic [1]. Aside from social distancing, benefits of virtual consultations include potential cost savings and support of sustainability.

Postpandemic, a universal return to face-to-face consultations may risk the loss of some of the advantages of telemedicine. However, rapid implementation and adoption without robust evaluation of usability, efficacy, and effectiveness could potentially lead to suboptimal health outcomes and downstream challenges to providers.

Guidance documents have been published to assist health care professionals to deliver telemedicine [2]. Reviews and evaluations to date have typically focused on specific condition groups and modalities, which does not reflect the variety often encountered in general pediatric outpatient services. Furthermore, there is significant variation in the quality and modality of telemedicine intervention evaluations and potential gaps in outcome measures [3]. A recent broader systematic review of randomized controlled trials (RCTs) in pediatric telemedicine [4] evaluated feasibility, accessibility, satisfaction, and outcomes but did not assess the evaluation of the telemedicine intervention.

Frameworks to benchmark and improve digital health interventions have been developed, for example by the National Institute for Health and Care Excellence (NICE) [5]. These are often designed to evaluate mature interventions and facilitate procurement decisions. In contrast, a World Health Organization (WHO)–published guidance [6] on monitoring and evaluating digital health interventions provides a more fluid framework that can also be applied to novel interventions undergoing iteration as well as more mature interventions being scaled up.

To this end, a group of clinicians with an interest in child health (the Child Health in Practice Group, a voluntary network of UK-based pediatricians) [7] highlighted the need to undertake a high-level scoping review of telemedicine interventions in pediatric outpatient care. The need to understand how children and young people can be effectively supported by emerging technologies was also an outcome finding of the Royal College of Paediatrics and Children Health (RCPCH) 'Paediatrics 2040' project [8]. This review aimed to assess if telemedicine interventions are being evaluated in line with international WHO guidance as well as if there is sufficient evidence to support postpandemic utilization of telemedicine in pediatric settings.

## Methods

### Study Rationale

The review was conducted in accordance with the Preferred Reporting Items for Systematic Reviews and Meta-Analyses extension for Scoping Reviews (PRISMA-SR) statement [9].

At initial study conception, 4 common conditions were examined with the aim to inform clinicians on the suitability of virtual care solutions in outpatient pediatric services. Diabetes was selected for readily measurable biomarkers that can track both long- and short-term disease control (eg, glycated hemoglobin [HbA1c] and blood glucose concentrations), asthma was selected because its biomarkers (eg, peak flow) predominantly reflect short-term disease control, epilepsy is a common condition without a clearly trackable biomarker, and nephrology [10] is an example of a less common patient population whose care is delivered in fewer specialist centers that cover large geographical areas. Initially intended as 4 separate reviews, early in this process, it became apparent that the similarity in methodologies, the number of resulting studies, and the telemedicine modalities in these studies meant that it was more practicable to report findings collectively.

### Eligibility Criteria

Studies were selected on the basis of the inclusion and exclusion criteria in Textbox 1.

**Inclusion and exclusion criteria, with rationale, of the studies.**

Include:

- Children (0-18 years) receiving outpatient care for any of the following: diabetes, asthma, epilepsy, or renal disease
- Interventions: telemedicine, remote consultation, video call, remote patient monitoring (collecting patient data outside of traditional health care settings to support ongoing patient care)
- Studies comparing interventions with usual outpatient care (including control arms, historic comparisons, or based on user with or without clinician perceptions)
- Studies published between 2011 and the present day
- Studies published in the English language
- Studies that are primary research studies

Exclude:

- Adults (>18 years) or where not possible to disaggregate data for participants younger than 18 years
- Outcome data pertaining to conditions other than diabetes, asthma, epilepsy, or renal disease
- Interventions not intended to replace current outpatient medical health care services:
  Education
  Behavioral interventions (eg, cognitive behavioral therapy)
  Family therapies (where not intended to replace current outpatient health care services)
- Remote patient monitoring not directly related to outpatient care:
  Support telemetry of electroencephalogram (EEG), electrocardiogram (ECG), or other continuous data from inpatients or transport patients
  Teleconferencing between health care professionals (eg, tertiary center reviews or multidisciplinary team meetings)
- Studies not published in the English language
- Studies that are conference abstracts, letters, study protocols, systematic reviews, or review articles.

### Information Sources and Search Strategy

PubMed, Embase, and CINAHL databases were searched using the search strategies presented in Table S1 in Multimedia Appendix 1 on April 15, 2021, and repeated on May 31, 2022.

### Selection Process

The Rayyan [11] web-based tool was used to assist the selection process, initially by identification of duplicates, which required acceptance or rejection by a reviewer. After removal of duplicates, all records were initially screened by title and abstract, performed independently by 2 reviewers assigned to each record. Decisions were unblinded after completion. Where decisions conflicted, this was resolved by discussion or a third reviewer if agreement could not be reached.

### Data Collection Process

Data were initially extracted into synthesis tables for each disease group and corroborated by a second reviewer. To ensure good inter-reviewer consistency, a blinded calibration exercise was performed.

### Data Items (Outcomes)

For all studies, the following data fields were extracted into the synthesis table:

- Title, author, date of publication, URL, and DOI
- Study design, number of participants, study population, and location of study
- The telemedicine intervention under evaluation and its maturity
- Evidence of impact on pediatric care in 3 domains:
  Usability and feasibility of telemedicine in pediatric settings
  Efficacy and effectiveness as evidenced by process and clinical outcomes [12]
  Implementation science issues
- Confidence in the strength of the evidence in each of these domains

Studies were analyzed for maturity of intervention, risk of bias, and outcomes reported. The WHO guidance [6] was used to determine the maturity of the telemedicine intervention, in turn defining the appropriate focus of evaluation as well as appropriate claims regarding the anticipated benefits of the intervention. Intervention maturity was defined by the size of deployment, intervention setting (controlled/uncontrolled), and what previous testing of the intervention has taken place. Data categories related to the impact on pediatric care were also based on the stages of evaluation outlined in the WHO guidance [6].

Due to the variety of study designs anticipated, formal quality assessment tools (eg, Critical Appraisal Skills Programme or National Institutes of Health National Heart, Lung, and Blood Institute tools) were not used. Instead, the hierarchy of evidence outlined in the WHO guidance [6] was used as a high-level indication of the confidence in the strength of the evaluations’ evidence, categorized as poor, fair, good, or excellent based on the overarching study methodology. This framework was chosen as it enables high-level assessments of evidence across different study designs and meaningful comparison of interventions of different maturities.

The blinded calibration exercise identified that proportions of agreement for both intervention maturity assessment and strength of evidence were 100% for all extractors.

Study outcome measures and findings were reviewed, collated, and synthesized in tabular form. Studies were examined for reported outcomes (feasibility and usability, clinical and process outcomes, and implementation science issues), and coded as either positive, negative, or equivocal.

## Results

**Figure 1:**
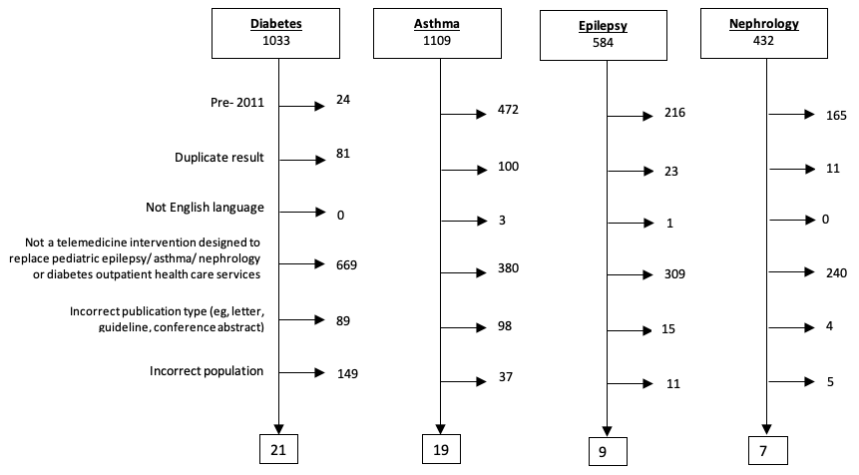
A series of 4 CONSORT diagrams reporting identified articles and reasons for exclusion; a total of 56 articles was suitable for final inclusion and analysis.

Following a priori exclusions (Figure 1), a total of 56 published studies relating to telemedicine in pediatric services were included.

### Description of Studies

A total of 11 studies were RCTs, of which 3 were multisite, the highest level in the WHO hierarchy of evidence [6]. Other study designs were quasiexperimental studies (9 studies), cohort studies (11 studies), case control studies (4 studies), cross-sectional studies (19 studies), observational studies (1 study), and quality improvement project (1 study).

Of these studies, 49 were quantitative in nature, 3 studies utilized mixed methods, and 4 studies were qualitative.

### Interventions and Stage of Maturity

The scale and maturity of the telemedicine deployments varied from prototypes undergoing user testing all the way to large-scale deployments covering multiple sites. Using the WHO 6-stage intervention maturity life cycle [6], the interventions across the primary papers were categorized as prototype or pre-prototype (9 studies), pilot (usually a single deployment in controlled circumstances; 42 studies), demonstration (moderate-scale implementation no longer in controlled settings; 3 studies), and scale-up (intervention that is ready for implementation at subnational or higher level; 2 studies).

A range of telemedicine modalities were utilized, with 46 studies examining a single modality, namely videoconferencing or video calls (22 studies); telephone (4 studies); instant messaging, chatbot, or SMS (3 studies); and remote patient monitoring (RPM; 17 studies). In 6 studies, the RPM platform was hosted on a smartphone application. The remaining 10 studies examined a combination of 2 or more of the modalities.

**Figure 2:**
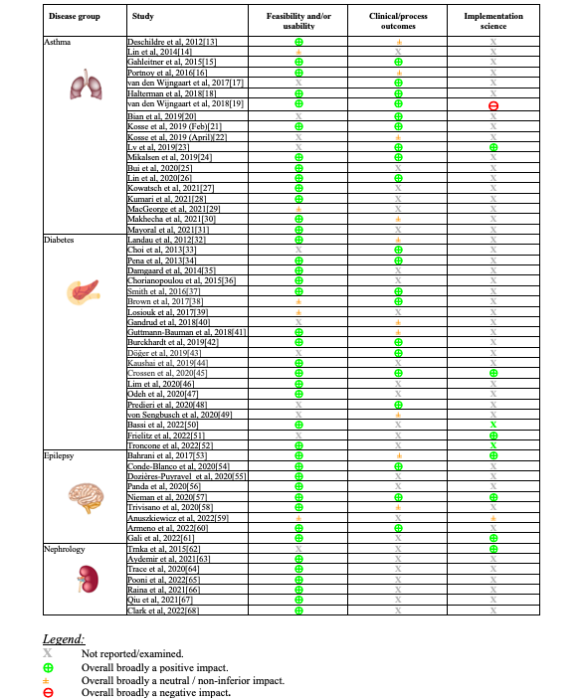
Summary of findings for main evaluation domains, classified as "Not reported," "Positive," "Neutral," or "Negative" [13-68].

Main outcomes were feasibility or usability, clinical or process outcomes, and implementation science issues. Figure 2 summarizes the data that were reported and overall findings. Clinical or process outcomes are included only where comparison was made and not if simply reporting the number of events, for example.

### Feasibility and Usability of Telemedicine

Data on feasibility or usability of the telemedicine intervention were reported in 45 studies. All but 1 of the studies not reporting these data looked at a pilot stage intervention, with the remaining study [20] examining a scale-up stage intervention. Technical difficulties were reported in 20 studies, but not all positively or negatively testified to this. A breakdown of the confidence in the strength of the evidence in this evaluation domain across the studies is outlined in Table 1.

**Table 1:** Summary of confidence in the strength of the evidence of usability or feasibility of telemedicine interventions across each condition group in 45 studies.

Variables		Epilepsy	Nephrology	Diabetes	Asthma	Total
**Confidence in the strength of the evidence, number of studies**						
	Excellent	0	0	0	1	1
	Good	3	0	10	11	24
	Fair	6	6	5	3	20
	Poor	0	0	0	0	0
Number of studies within condition group reporting these data, n (%)		9/9 (100)	6/7 (86)	15/21 (71)	15/19 (79)	45/56 (80)

In the results, there were common positive themes identified from health care professionals, patients, and families in support of the telemedicine interventions, including convenience, perceived cost savings, and ease of use. Interventions were generally well-accepted and would be recommended to others. Use in preference to usual care was explored in 4 studies. In 1 study [63], families reported use of telehealth beyond the coronavirus pandemic was not wanted, and in 3 studies [50,64,68], parents emphasized it was wanted to supplement but not substitute in-person clinics.

There were 16 studies (16/56, 29%) that had requirements for participants to have specific owned mobile devices or internet access or excluded participants for whom technical issues meant consultation could not proceed. Damgaard and Young [35] presented a statistically significant improvement in parental satisfaction following intervention; however, broadband bandwidth was insufficient for many schools with the intervention, necessitating installation of separate internet connections.

For smartphone-based RPM, this evaluation domain was conducted in 4 of 6 studies. In 1 study [21], 96% of pharmacists were satisfied with the intervention, and although 77% of patients felt it was easy to use and 78% would recommend to others, 19% reported technical issues as a reason to not use the intervention. In 3 other studies, patient satisfaction was reported, but health care professionals’ satisfaction was not. In 1 pilot study [25], children were satisfied (63%) or very satisfied (32%) with their experience with the app, similarly rated by parents. The interventions by Mikalsen et al [24] had a median score of 18/20 for functionality and overall assessment, while mean System Usability Scale scores in the study by Mayoral et al [31] were 92.9 (0: negative; 100: positive).

The majority of evidence against the use of telemedicine came from studies in asthma services, although this is in the context of otherwise very positive evidence, including from 1 study [13] with excellent confidence in the strength of the evidence. Prototype interventions were examined in 2 studies [14,15], with issues related to the usability of the non-smartphone–based remote monitoring system. A third study [19] looking at scale-up of a non-smartphone–based remote monitoring system found that 4 of 14 sites were unable to successfully implement the intervention, although in only 1 case was a reason (insufficient staff) provided. Finally, a pilot study [28] of telephone consultations for asthma found that only 40% of respondents wished to continue with the modality beyond the pandemic.

### Telemedicine Impact on Processes and Clinical Outcomes

Clinical and process outcome data were collected in 31 studies: 23 pilot studies, 4 prototype studies, 2 scale-up studies, and 2 demonstration studies. Of those not reporting outcome data, 19 were pilot studies, 5 were prototype studies, and 1 was a demonstration.

For the studies that collected and reported outcomes data, a breakdown of the confidence in the strength of this evidence is outlined in Table 2*.*

Outcome data, when provided, were mostly, but not universally, positive. Process measures were more frequently provided than clinical outcomes. Almost all the studies with good or excellent confidence in the strength of the evidence looked at asthma or diabetes.

**Table 2:** Summary of confidence in the strength of the evidence of clinical or process outcomes of telemedicine interventions across each condition group in 30 studies.

Variables		Epilepsy	Nephrology	Diabetes	Asthma	Total
**Confidence in the strength of the evidence, number of studies**						
	Excellent	0	0	1	2	3
	Good	1	0	8	9	18
	Fair	4	0	1	0	5
	Poor	0	0	2	2	4
Number of studies within condition group reporting these data, n (%)		5/9 (56)	0/7 (0)	12/21 (57)	13/19 (68)	30/56 (54)

A summary of the outcomes, appraisal, and quality of evidence of each of the 56 studies is presented in Table S2 in Multimedia Appendix 1 [13-68]. Across all subgroups, the telemedicine intervention was acceptable or feasible to the patient, their family, or the health care professionals; however, not all studies reported the view of all parties. Telemedicine interventions were considered beneficial, and for diabetes and asthma particularly, perceived benefits included improved understanding and management of the child’s condition, with some studies reporting measures of quality of life. Across all studies, there was limited measurement of standardized clinical outcomes, although these were more commonly reported in asthma (peak flow) and diabetes (HbA1c). Many studies reported interventions adopted as a consequence of the COVID-19 pandemic, and although generally well-accepted in their own right, acceptability in comparison or instead of usual care was not always explored. The study by Gandrud et al [40] was the only study in which clinical outcome data were compared with standard care. Their intervention demonstrated improvement in HbA1c and health-related quality of life in the intervention group, but this was not statistically significant.

One limiting factor common across all groups was exclusions of participants based on lack of access to the internet or an appropriate device.

### Confidence in the Strength of the Evidence of Studies

**Figure 3:**
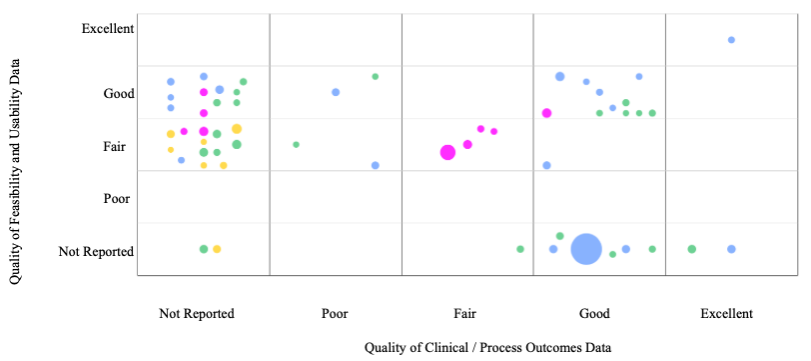
Bubble plot depicting the quality of data (per World Health Organization guidance [6]) of studies reporting feasibility and/or usability and outcomes. The size of the bubbles corresponds to the size of the study cohort, and the color indicates the disease category: green: diabetes; blue: asthma; pink: epilepsy; yellow: nephrology.

As outlined in Figure 3, 10 studies reported outcome data without examining feasibility or usability, while 23 studies only reported feasibility or usability data. The majority (21/22, 96%) of studies that reported both variables had fair or good confidence in the strength of findings. A solitary study [13] was assessed as having the highest confidence (excellent) for both evidence of feasibility or usability and evidence on outcomes.

### Implementation Science Issues of Telemedicine

The most common implementation science factor examined was cost-effectiveness, reported in 5 studies (5/56, 9%; details in Table S2 in Multimedia Appendix 1).

One study [53] examining epilepsy services focused on a demonstration of a telephone consultation intervention that identified a saving of 865 INR (approximately US $11.25 per patient). A study in the pilot phase [62] that utilized multiple telemedicine interventions in nephrology patients, identified cost savings, estimated to be US $505 per consultation, mostly associated with reducing travel and accommodation requirements. Finally, a pilot study [23] of a smartphone-based RPM intervention for asthma care found a statistically significant decrease in medical expenses from 1179 RMB to 931 RMB (approximately US $145) per patient. A quasirandomized multicenter study found direct diabetes-associated 6-month costs to be €4702 in the intervention group, compared with €4936 in the control group [51]. Another pilot study [61] found a statistically significant difference (*P*<.001) in out-of-pocket expenses for telehealth, US $35, compared with US $176 for an in-person visit.

When compared with usual clinics, Gali et al [61] found that, with telehealth, missed school hours were reduced by 49%, missed work hours were reduced by 48%, and mileage was 32 miles compared with 49 miles for in-patient visits (all *P*<.001). Significant time savings were also identified when using videoconferencing for diabetes [45] and reported (but not quantified) by 98% of respondents using non-smartphone–based remote monitoring for epilepsy [57]. The final implementation science issue identified was in non-smartphone remote monitoring for asthma, for which lack of structural financial reimbursement was identified as the main barrier [19].

## Discussion

### Principal Findings

This review examined the existing literature regarding pediatric telemedicine interventions in asthma, diabetes, epilepsy, and nephrology. These conditions cover a mixture of common and rare diseases, with and without availability of biomarkers including short- and long-term control. Reported clinical outcomes were heterogeneous, making pooling of results impossible. A wide variety of outcome and process measures was used, with no clear standardization, even within condition groups. This also made cost-effectiveness analysis challenging. Although several studies did report cost-effectiveness, this was usually heavily caveated and thus not conducive to supporting larger commissioning or service redesign decisions.

With regards to how digital interventions are used and perceived, the majority of identified studies examined the evidence on usability and feasibility of the telemedicine intervention. Satisfaction levels among clinicians and patients were high, with users open to and often enthusiastic for future use of telemedicine. The confidence in the strength of these findings was most typically fair or good. In no study were there observed disagreements between the sentiment of professionals and those of patients and families. Further research should build on this evidence base, and we recommend that research and evaluation frameworks encourage the standardized collection of usability and acceptability data, particularly in prototype, pilot, and demonstration stage interventions.

Where problems with the usability and feasibility of telemedicine were identified, this was primarily due to technical issues with video conferencing as well as non-smartphone–based RPM. In one instance [19], wider implementation issues (eg, lack of staff) prevented successful scale-up of an asthma non-smartphone–based RPM intervention in 4 of 14 sites and identifies lack of structural financial reimbursement of web-based monitoring as a significant obstacle in diffusion of eHealth innovation. Such challenges of large-scale digital implementation are well known, with national reports suggesting that the process can take several years to fully iterate [69]. In contrast, smartphone-based RPM had particularly positive evidence, with clinician and patient satisfaction levels over 90% and good confidence in the strength of the evidence in one-half of these studies.

With regards to the impact of digital interventions, the majority of literature found that outcomes improved or were equivocal to traditional care. Where there was evidence of negative impact on clinical outcomes, this was not statistically significant. The strength in the confidence in clinical outcome evidence varied across the literature. There was poor evidence in nephrology. In contrast, in diabetes and asthma, conditions with established biomarkers that can objectively monitor disease progression, there were several studies with excellent confidence in the evidence. Unfortunately, this was not always coupled with good quality evidence on the perception and use of the intervention. Only 18 studies reported good or excellent confidence in the evidence for both how the studied intervention was received and its impact.

The potential long-term benefits of telemedicine include decreased travel (time and expense) for families, reduced exposure to nosocomial infection for vulnerable patients, and reductions in the carbon footprint of health care [70]. The COVID-19 pandemic has seen the rapid implementation of telemedicine interventions with many of these benefits realized over the past 2 years [1]. Improvements in digital implementation could represent a boon for its wider adoption, but caution should prompt the collection of meaningful data to ensure these are noninferior to traditional modalities of care.

Among the potential limitations are a lack of physical examination and the resulting impact on clinical decision-making, privacy concerns, and the impact of digital exclusion [63]. However, reassuring outcome data and user satisfaction, in line with WHO guidance [6], can provide assurance in these regards. One important reflection is the lack of child protection or safeguarding literature in the area [3].

### Evidence From Previous Literature

The search protocols identified 5 previous reviews [71-75] focused on telemedicine in diabetes and not included in the final synthesis. These papers examined a variety of modalities including video consultations, telephone consultations, text services, and RPM, all in the pilot stage. Of these reviews, 4 [71,73-75] identified data on feasibility and usability, with findings consistent with this synthesis. With the exception of a small number of technical problems with 1 intervention’s GPRS wireless system [71], findings were universally positive. Identified benefits included improved access to care, increased parental satisfaction, and perceived time savings.

Four reviews identified outcome data [71-74], 2 of which reported statistically significant improvements in HbA1c and emergency department visits [72,74]. Two reviews noted that telephone, SMS services, and non-smartphone–based RPM [71,73] had no impact on HbA1c. One of these reviews [71] identified that telephone and SMS services may improve patient engagement and self-efficacy.

### Recommendations

The review identifies an urgent need for a more standardized approach to evaluation of digital interventions. There is a lack of literature examining this area despite the increasing adoption of such virtual consultations. Much of the literature does not include meaningful data on usability and feasibility of the intervention recommended by the WHO guidance [6] and particularly important for early, pilot-stage interventions.

Although usability is an important measure, it is also important to evaluate changes in health care practices for noninferiority for clinical outcomes. Evaluations should be designed to review meaningful clinical outcomes, for example, in pediatric nephrology, the rate of progression of renal impairment and transplant survival. Proxy measures for these could also include proteinuria and medication concordance. Standardization, perhaps through an agreed outcome set, would enable interventions to be compared and results pooled. Professional organizations such as the RCPCH can lead this to produce evaluation frameworks, facilitating scientific rigor among suppliers to undertake high-quality evaluations.

For clinicians interested in digital implementation, the early findings across these studies are promising, particularly in smartphone-based RPM and video consultation. Clinicians who are implementing or piloting digital interventions should focus on building robust evaluation strategies, in line with established guidance. Additions to the evidence base should focus on promoting higher-quality studies, ideally RCT or other experimental study designs.

High-quality evaluation can be promoted through restructuring innovation funds, which should reward comprehensive evaluation strategies aligned with international guidance and which should ideally set aside a portion of funding to be used exclusively for evaluation.

### Limitations

Although the review is a starting point for further evaluation and research, a number of limitations should be acknowledged. The focus on 4 disease groups, a pragmatic amalgamation of initially parallel reviews, provides breadth but is not complete. The review is also at high risk of publication bias. Although we found several examples of prototype interventions with mixed results, other interventions that may have had unfavorable results may have been excluded from publication. A common theme in studies was the potential exclusion of participants who did not have access to appropriate technology, and this may have implications of health inequalities in this space.

The WHO framework [6] offers a high-level assessment of digital health interventions, enabling comparison between different study designs. However, it does not differentiate between the quality of similar designs. For example, a poorly designed multisite RCT may offer less compelling evidence than a well-designed longitudinal study, which would not be reflected in our chosen approach. Nonetheless, the framework is well-suited to assess interventions of varying maturity and enables some comparison between different study designs.

### Conclusion

Current evidence indicates that, across a range of modalities, including telephone or video calls, text messaging, and more comprehensive RPM systems, telemedicine is viewed as an acceptable tool to deliver pediatric outpatient care. Although promising, existing results should be taken with consideration of the data’s limitations. When telemedicine interventions are to be implemented, appropriate gathering of data is needed to secure an evidence base that interventions are safe and not associated with inferior clinical outcomes. Outcome measures should include child safety and clinical outcomes to ensure noninferiority to traditional face-to-face consultation.

## Appendix

Multimedia Appendix 1 Supplementary tables.

## Abbreviations

HbA1c: glycated hemoglobin
NICE: National Institute for Health and Care Excellence
PRISMA-SR: Preferred Reporting Items for Systematic Reviews and Meta-Analyses extension for Scoping Reviews
RCPCH: Royal College of Paediatrics and Child Health
RCT: randomized controlled trial
RPM: remote patient monitoring
WHO: World Health Organization
