# Supplementary material for: Use of Telemedicine in Pediatric Services for 4 Representative Clinical Conditions: Scoping Review
Source: J Med Internet Res. 2022 Oct 26;24(10):e38267. doi: 10.2196/38267 (PMC9647449; doi:10.2196/38267)
Supplement: Multimedia Appendix 1 [file jmir_v24i10e38267_app1.docx]

**Table S1.** Table of the search strategy used in each database for each group.

| **Asthma Search Strategy - performed 15/04/2021** |
| --- |
| **PUBMED:**  (("Remote Consultation"[Mesh]) OR "Telemedicine"[Mesh]) AND (("Child"[Mesh]) OR "Child, Preschool"[Mesh]) OR "Pediatrics"[Mesh])) AND ("Asthma"[Mesh]) |
| **CINAHL PLUS:**  (video conferencing OR video call OR video communication OR video conference OR video consultations OR video appointments OR video consultation OR video calls OR skype OR video chat OR facetime OR video chat OR video calling OR telehealth OR telemedicine OR telemonitoring OR telepractice OR telenursing OR telecare OR telecommunication OR remote consultation OR remote healthcare OR remote health) AND (children OR adolescents OR youth OR child OR teenager OR childhood OR paediatric OR pediatric OR paediatrics OR pediatrics OR children OR child OR young person OR paediatric patient OR pediatrics OR infant OR adolescent) AND (Asthma) |
| **EMBASE:**  (children OR adolescents OR youth OR child OR teenager OR childhood OR paediatric OR pediatric OR paediatrics OR pediatrics OR children OR child OR young person OR infant OR paediatric patient OR adolescent OR pediatric patients) AND (video conferencing OR video call OR video communication OR video conference OR video consultations OR video appointments OR video consultation OR video appointment OR video calls OR skype OR video chat OR facetime OR video conference OR video conferencing OR video calling OR telehealth OR telemedicine OR telemonitoring OR telepractice OR telenursing OR telecare OR telecommunication OR remote consultation OR remote healthcare OR remote health) AND (Asthma) |
| **Diabetes Search Strategy - performed 15/04/2021** |
| **PUBMED:**  (("Remote Consultation"[Mesh]) OR "Telemedicine"[Mesh]) AND (("Child"[Mesh]) OR "Child, Preschool"[Mesh]) OR "Pediatrics"[Mesh])) AND ("Diabetes Mellitus"[Mesh]) |
| **CINAHL PLUS:**  (video conferencing OR video call OR video communication OR video conference OR video consultations OR video appointments OR video consultation OR video calls OR skype OR video chat OR facetime OR video chat OR video calling OR telehealth OR telemedicine OR telemonitoring OR telepractice OR telenursing OR telecare OR telecommunication OR remote consultation OR remote healthcare OR remote health) AND (children OR adolescents OR youth OR child OR teenager OR childhood OR paediatric OR pediatric OR paediatrics OR pediatrics OR children OR child OR young person OR paediatric patient OR pediatrics OR infant OR adolescent) AND (Diabetes) |
| **EMBASE:**  (children OR adolescents OR youth OR child OR teenager OR childhood OR paediatric OR pediatric OR paediatrics OR pediatrics OR children OR child OR young person OR infant OR paediatric patient OR adolescent OR pediatric patients) AND (video conferencing OR video call OR video communication OR video conference OR video consultations OR video appointments OR video consultation OR video appointment OR video calls OR skype OR video chat OR facetime OR video conference OR video conferencing OR video calling OR telehealth OR telemedicine OR telemonitoring OR telepractice OR telenursing OR telecare OR telecommunication OR remote consultation OR remote healthcare OR remote health) AND (Diabetes) |
| **Epilepsy Search Strategy - performed 15/04/2021** |
| **PUBMED:**  (("Remote Consultation"[Mesh]) OR "Telemedicine"[Mesh]) AND (("Child"[Mesh]) OR "Child, Preschool"[Mesh]) OR "Pediatrics"[Mesh])) AND ("Epilepsy"[Mesh]) |
| **CINAHL PLUS:**  (video conferencing OR video call OR video communication OR video conference OR video consultations OR video appointments OR video consultation OR video calls OR skype OR video chat OR facetime OR video chat OR video calling OR telehealth OR telemedicine OR telemonitoring OR telepractice OR telenursing OR telecare OR telecommunication OR remote consultation OR remote healthcare OR remote health) AND (children OR adolescents OR youth OR child OR teenager OR childhood OR paediatric OR pediatric OR paediatrics OR pediatrics OR children OR child OR young person OR paediatric patient OR pediatrics OR infant OR adolescent) AND (Epilepsy) |
| **EMBASE:**  (children OR adolescents OR youth OR child OR teenager OR childhood OR paediatric OR pediatric OR paediatrics OR pediatrics OR children OR child OR young person OR infant OR paediatric patient OR adolescent OR pediatric patients) AND (video conferencing OR video call OR video communication OR video conference OR video consultations OR video appointments OR video consultation OR video appointment OR video calls OR skype OR video chat OR facetime OR video conference OR video conferencing OR video calling OR telehealth OR telemedicine OR telemonitoring OR telepractice OR telenursing OR telecare OR telecommunication OR remote consultation OR remote healthcare OR remote health) AND (Epilepsy) |
| **Nephrology Search Strategy - performed 15/04/2021** |
| **PUBMED:**  (("Remote Consultation"[Mesh]) OR "Telemedicine"[Mesh]) AND (("Child"[Mesh]) OR "Child, Preschool"[Mesh]) OR "Pediatrics"[Mesh])) AND ("Nephrology"[Mesh]) |
| **CINAHL PLUS:**  (video conferencing OR video call OR video communication OR video conference OR video consultations OR video appointments OR video consultation OR video calls OR skype OR video chat OR facetime OR video chat OR video calling OR telehealth OR telemedicine OR telemonitoring OR telepractice OR telenursing OR telecare OR telecommunication OR remote consultation OR remote healthcare OR remote health) AND (children OR adolescents OR youth OR child OR teenager OR childhood OR paediatric OR pediatric OR paediatrics OR pediatrics OR children OR child OR young person OR paediatric patient OR pediatrics OR infant OR adolescent) AND (nephrology OR renal OR kidney disease) |
| **EMBASE:**  (children OR adolescents OR youth OR child OR teenager OR childhood OR paediatric OR pediatric OR paediatrics OR pediatrics OR children OR child OR young person OR infant OR paediatric patient OR adolescent OR pediatric patients) AND (video conferencing OR video call OR video communication OR video conference OR video consultations OR video appointments OR video consultation OR video appointment OR video calls OR skype OR video chat OR facetime OR video conference OR video conferencing OR video calling OR telehealth OR telemedicine OR telemonitoring OR telepractice OR telenursing OR telecare OR telecommunication OR remote consultation OR remote healthcare OR remote health) AND ((nephrology or renal or chronic kidney disease or CKD).mp) |

**Table S2.** Table of results summarizing outcomes, appraisal, and quality of evidence for all papers included in the analysis.

|  | **Study** | **Study size: study design** | **Intervention: *stage of maturity*** | **Inclusion Criteria** | **Exclusion Criteria** | **Feasibility and/or Usability Outcomes to** | | **Strength of findings** | **Strength of Findings. Process or Clinical Outcome data** | **Strength of findings. Implementation science issues** | **Headline Findings** |
| --- | --- | --- | --- | --- | --- | --- | --- | --- | --- | --- | --- |
|  |  |  |  |  |  | **Service User(s)** | **Clinician** |  |  |  |  |
| **Asthma** | Deschildre et al, 2012^13^ | 44: RCT | RPM without smartphone app: *Pilot* | 1. 6–16 year old  2. Severe allergic asthma. | Congenital or acquired chronic illnesses other than asthma. | Parents were satisfied with the monitoring of respiratory function (57.1% very satisfied and 33.1% satisfied versus 9.5% not satisfied), and felt reassured (57.1% very reassured and 42.9% reassured). Parents believed that the monitoring was generally easy to carry out (57.1% thought it very easy and 38.1% easy versus 4.8% who thought it was not easy). | N/A | Excellent | **Excellent.** No significant difference in exacerbations, days of systemic steroid use, unscheduled visits or hospitalisations. No significant difference in inhaled steroids, QoL, improvement in clinical scores, FEV1/FEF25-75. | N/A | This RCT found no evidence that daily home telemonitoring of FEV1 was superior to conventional management in terms of lung function, quality of life or steroid treatment, despite the additional effort required by young people to perform daily spirometry. Only 35 patients (out of 225) were able to complete a full year. However, parents evaluated the intervention very positively, which highlights the benefit of the educational aspect. The authors argue that overall the evidence is not supportive of intensive telemonitoring. |
|  | Lin et al, 2014^14^ | 41: Quasi-experimental study | RPM without smartphone app: *Prototype* | 41 voluntary asthmatic children (and their parents) from Taiwan were intervened to examine the proposed system. | None described | 40-50% of users completely agreed with intervention's functions. 5-10% did not feel the intervention was convenient. Some children felt uncomfortable when finishing the diary with parents. The system was deemed feasible to help self-management and monitoring health conditions but required upgrading to a more user friendly interface. The average scores were scaled in each domain demonstrated the feasibility of the proposed system. | Healthcare professionals were able to get a more detailed picture of patients' morbidity because of real-time online analytical and diagrammatical reports. | Good | N/A | N/A | This web-based remote monitoring system was found to have performed well. Acceptability rates across 7 domains were 88% or higher. The authors concluded that participants would like to adopt the intervention for daily disease management, and that physicians valued having real-time data and analysis. Suggestions for improvements were provided by young people, parents and professionals, this included a more user-friendly interface and making the platform mobile-friendly. |
|  | Gahleitner et al, 2015^15^ | 32: Cohort study | RPM without smartphone app: *Prototype* | 1. 5–11 year old with doctor diagnosed asthma  2. Treatment with inhaled corticosteroids  3. History of interval symptoms and exacerbations with upper respiratory tract infections (at least two such exacerbations in the previous year). | 1. Unable to perform lung function measurements  2. Poor compliance with medication  3. Treated with more than 1,000 mcg beclomethasone equivalent a day  4. History of other lung diseases | All (22/22) parents were “very happy” or “happy” to use the SMS. Of the 20 parents completing the follow up questionnaire, 95% (19/20) found the system user-friendly and 55% (11/20) would be more likely to participate in studies if they were using SMS data collection (none less likely). 25% (5/20) were “sometimes unhappy” about receiving messages. Reasons included “lack of time” or being “at work”. 2 parents reported issues with phone signal. | Real-time capture of data is well accepted and addressed some of the pitfalls of backfilled paper diaries. There were, however technical issues - correctly reported PEFR values were found in 65.5% of all cases. In 8.3% PEFR values sent were “self-invented” and 2.4% of values were missing. | Good | **Good.** Mean (SD) “SMS-diary and PEFR-meter compliance” were 96% (8) and 84% (21) during baseline and 91% (12) and 82% (20) during illness respectively. | N/A | The authors argue that real-time SMS diary is well accepted and avoids some of the issues of paper diaries. The authors argue that many of the technical limitations identified in this study could be overcome with better user interfaces and integrated devices. |
|  | Portnoy et al, 2016^16^ | 169: Case control study | Videocall or videoconference with devices: *Pilot* | 1. Scheduled appointment for asthma in the Children’s Mercy Hospital allergy clinic  2. Previous diagnosis of asthma or if the reason for their visit included asthma. | Not included if patients did not reside near St Joseph, MO, or Wichita, KS. | 47/59 strongly agreed that telemedicine was as good as in person, 7 mostly agreed and 4 were neutral. 51/58 recommended healthcare appointments by telemedicine, 4 mostly agreed, 3 neutral. Only 1 patient disagreed that distance travelled (which less than would have been the case for in-person care) was acceptable. Only 1 patient expressed that not having to travel was not important to them. | N/A | Fair | **Good.** Of 169 children, 100 were seen in-person and 69 via telemedicine. A total of 34 in-person and 40 telemedicine patients completed all 3 visits. All had a small, although statistically insignificant, improvement in asthma control over time. Telemedicine outcomes were noninferior to in-person visits. | N/A | Case-control study evaluating asthma outcomes between a telemedicine group and an in-person group. Some patients did show up to the first visit but decided to not complete the surveys after they were seen. There were more dropouts in the control group, who had to travel to appointments. There was no significant difference in the Adjusted Asthma Scores (AASs) between telemedicine and control groups after 30 days and again after 6 months. Most patients from the telemedicine group strongly agreed that it was easy to see, hear and understand the health care professional. They also had a sense that the health care professional cared and that they could ask questions. Most agreed that they would recommend telemedicine. |
|  | van den Wijngaart et al, 2017^17^ | 210: RCT (multicentre) | RPM without smartphone app: *Pilot* | 1. 6–16 years old  2. Doctor’s diagnosis of asthma based on symptoms and a bronchodilator response of FEV1 % pred >9%, and/or airway hyperresponsiveness, and/or signs of eosinophilic airways inflammation (elevated FENO >25 ppb). | 1. ICU admission for asthma in the preceding 5 years 2. Difficult-to-treat asthma 3. Use of omalizumab 4. Other chronic diseases 5. Parents or children unable to read and understand Dutch. | N/A | N/A | N/A | **Good.** For both groups, a statistically significant difference in the number of symptom free days was demonstrated after 16 months, in favour of the virtual asthma clinic (VAC) compared with usual care. At 16 months, there was also a significant difference in mean childhood asthma control test scores of 1.17 points (95% CI 0.09–2.25; p=0.03) in favour of VAC. In children 12–16 years of age, asthma control, FEV1, FENO, ICS daily dose, numbers of asthma exacerbations, telephone calls, unscheduled visits to the outpatient clinic or emergency department and hospital admissions was similar after 16 months of follow-up. | N/A | Multi-centre RCT comparing usual care with virtual asthma clinic. Statistically significant difference between the groups in favour of virtual asthma clinic for symptom-free days (1.23 days 95% CI 0.42-2.04; p=0.003); C-ACT (difference of 1.17 points, 95% CI 0.09–2.25; p=0.03). The usability and feasibility of the intervention was not formally evaluated. |
|  | Halterman et al, 2018^18^ | 400: RCT | Mixed. Video and telephone: *Pilot* | 1. Physician-diagnosed asthma with persistent symptoms or poor control based on National Heart, Lung, and Blood Institute guidelines  2. 3 - 10 years old  3. Enrolment in the Rochester City School District. . | 1. Unable to speak English 2. No access to a telephone for follow-up 3. Other significant medical conditions (eg, congenital heart disease or cystic fibrosis) that might interfere with assessments | 95.7% of caregivers stated that the program was helpful. 96.5% said that they would be willing to participate in a similar program. | N/A | Good | **Good.** Postintervention, the school-based telemedicine enhanced asthma management programme (SB-TEAM) group had more symptom-free-day per 2 weeks (11.6 vs 10.97; difference, 0.69; 95% CI, 0.15-1.22; P = .01), fewer symptom days, symptom nights, and days with limited activity compared with children in the enhanced usual care (eUC) group. More children in the SB-TEAM group were prescribed a preventive asthma medication (91% vs 67%; odds ratio, 8.67; 95% CI, 4.19-17.95), and fewer had emergency department visits or hospitalizations for asthma (7% vs 15%, odds ratio, 0.52; 95% CI, 0.32-0.84) compared with children in the eUC group. Children in the SB-TEAM group had a greater decline (i.e. improvement) in FeNO level compared with children in the eUC group (mean difference, −5.54; 95% CI, −9.8 to −1.3). | N/A | RCT comparing enhanced usual care (eUC) to school-based telemedicine enhanced asthma management programme (SB-TEAM). Children in the SB-TEAM group had better improvements in disease control, fewer symptoms, and were more likely to have additional medications prescribed. Satisfaction with the program was high. |
|  | van den Wijngaart et al, 2018^19^ | 141: Cohort study | RPM without smartphone app: *Scale- Up* | 1. 6 of 8 hospitals in prior RCT approached and participated.  2. Additional 8 hospitals invited through email and participated. | None described | The majority (56/60, 93.3%) of the parents concluded that the virtual asthma clinic (VAC) had added value to current daily paediatric asthma management. Parents were satisfied with the attractiveness, usefulness, and user-friendliness of the innovation. Professionals experienced high patient satisfaction. Further, they stated that patients’ compliance, self-management, and knowledge were promoted by the use of the VAC. Security was no issue for 96% (58/60) of the patients or parents, as they trusted that personal information on the VAC was safe. The VAC was scored an 8 or higher (based on a 10-point scale, with 10 corresponding to maximum satisfaction) by 78% (47/60) of the parents. Similar scores were reported by the children completing the questionnaire. | Implementation unsuccessful in 4/14 hospitals. 1 due to insufficient staff, 3 no reason. Program felt to be user friendly but concerns about technology failure. Innovation was clear, easy, and user-friendly. Mixed views on impact on workload. Some professionals mentioned advantage of the intervention to substitute routine outpatients. However others felt that web-based monitoring was not an adequate substitution without technological integration and risks losing sight of patients. | Good | **Poor.** Parents concluded this led to better asthma control. | **Poor.** Whilst not quantified, observation made that the most important barrier for successful implementation of the VAC was the lack of structural financial reimbursement for Web-based monitoring. | This qualitative study identified several major perceived barriers and facilitators to the web-based remote monitoring portal's success. Implementation issues are complex and multifactorial, with no single factor identified as a key barrier or enabler. The study lacks more information that could have been ascertained from in-depth interviews, and the questionnaire used was not validated although no validated questionnaire existed at the time of the research. The authors have proposed a list of recommendations to support the implementation of digital health tools, which should be tailored to the needs of individuals and organisations. Overall, despite the challenges including lack of reimbursement and interoperability, professionals, parents and patients were all generally enthusiastic for the intervention and it has become part of regular care for children with asthma. |
|  | Bian et al, 2019^20^ | 23575: Case control study | Videocall or videoconference: *Scale-Up* | 1. 3 - 17 years old  2. Enrolled in South Carolina Medicaid at least 1 month  3. Lived in Williamsburg or any of its 4 neighbouring counties in 2012-17.  4. An asthma subsample that only included children with a diagnosis of comorbid asthma | None described | N/A | N/A | N/A | **Good.** Approximately 21% relative decrease in ED visits. The trend of the annual proportions of ED visits in Williamsburg decreased at a faster pace than that in the control counties during the postintervention period. The strength of the association increased over the postintervention period, becoming significant during the third year of the program: reduction of 1.11 (95% CI, −1.82 to −0.41; P < .01) percentage point per 100 children per month (or an approximately 35% relative decrease) during the third year. | N/A | Assessment of associations of a school-based telehealth programme on all-cause emergency department visits, with additional intervention and subsample analysis for asthma. Associated with a 21% relative decrease in ED visits, in asthma subgroup, compared with no association between intervention and attendances in whole group. Not specific to asthma and based on telehealth provided on an as needed basis, in schools only. |
|  | Kosse et al, 2019 (Feb)^21^ | 137: Cross-sectional study | Smartphone app: *Pilot* | All pharmacists and patients in the ADAPT study | None described | 76.6% of patients felt it was easy to use. 54.9% felt easier than contacting pharmacy. 50% felt it was useful. 78% would recommend to others. Main reasons to not use intervention (at all) were forgetfulness (50.0%; 27/54) and technical issues (18.5%; 10/54). | 95.7% of pharmacists were satisfied with the intervention. 69.6% felt it was user friendly. 95.8% of pharmacists who did not use it would like to use it in their pharmacy | Fair | **Poor.** 47.8% of pharmacists felt that the intervention improved medication adherence. 18% of patients reported being more aware of their medication and using their medication more regularly. | N/A | This was a cross-sectional evaluation of the ADolescent Adherence Patient Tool (ADAPT) intervention. Patients and professionals were positive about the intervention and professionals who did not have access to the intervention expressed an interest in adoption. Study participants felt that pharmacists were an appropriate professional to be involved in this intervention, reflecting current local practice on monitoring and management of asthma. Although the study is subject to response bias, it provides promising early findings which warrant further more generalizable study with a view to supporting the development of reimbursement guidelines and better embedment in workflows. |
|  | Kosse et al, 2019 (April)^22^ | 234: RCT | Smartphone App: *Pilot* | 1. 12–18 years old 2. Filling of at least two prescriptions for ICS or a fixed combination of ICS/LABA during the previous 12 months  3. Have a smartphone (iOS or Android). | 1. Insufficient comprehension of the Dutch language 2. Dependent on (in)formal carers to take their medication. | N/A | N/A | N/A | **Excellent.** No intervention effect on clinical scores, adherence, or asthma control across the population as a whole. There was observed improvements in adherence in previously poor adherent populations. | N/A | Cluster randomized control trial evaluating effectiveness of ADolescent Adherence Patient Tool (ADAPT tool - mHealth). The intervention had little effect on clinical scores, adherence, or asthma control for whole population, but improvement in adherence in those with low baseline Medication Adherence Report Scale (MARS) scores (MARS +2.52, p=0.02). No acceptability data. |
|  | Lv et al, 2019^23^ | 197: RCT (multicentre) | Mixed. Smartphone RPM and telephone: *Pilot* | 1. 6 - 12 years old  2. Medical history, symptoms and signs consistent with the diagnosis of asthma  3. Positive asthma predictive index  4. Willingness and ability to correctly use an inhaler  5. Possession of a smartphone and ability to use the mobile application  6. Ability to correctly use the Childhood Asthma Control Test (C-ACT). | 1. Severe asthma exacerbation at enrolment  2. Concurrent acute or chronic rhinosinusitis, obstructive sleep apnoea, or rheumatic diseases  3. Co-morbidities including bronchopulmonary dysplasia, bronchiectasis, tracheal or bronchial malacia, obliterative bronchiolitis, diffuse pan-bronchiolitis, or tuberculosis  4. primary immunodeficiency. | N/A | N/A | N/A | **Good.** The frequency of asthma exacerbations significantly decreased in the post-enrolment period in the two groups with a more significant decrease observed in the experimental group. The experimental group had a statistically significant better treatment adherence, Childhood Asthma Control Test (C-ACT) scores, diagnosed respiratory infections, days of antibiotic use, parental work loss and school absence than those in the control group. Between the two groups there was no statistically significant difference in the number of children who required oral steroid treatment or who stepped down their treatment. No patient required treatment step-ups during the study period. | **Good.** Medical expenses decreased significantly in the experimental group compared with those in the control group 1179 vs 931 Yuan (approximately $145) per year per patient | Well -designed multi-centre randomized clinical trial evaluating effectiveness of mHealth assisted nurse-led management model. Frequency of exacerbations decreased in both groups, with a greater decrease in the experimental group. Compared with the control group, secondary outcomes including adherence were all improved in the intervention group. Acceptability not explored. The authors comments that findings may have limited generalizability due to exclusion of non-Android software as well as users who did not have a smartphone. |
|  | Mikalsen et al, 2019^24^ | 22: Quasi-experimental study | Smartphone app: *Prototype* | 1. 5–12 years old with asthma  2. At least one parent with an iPhone  3. Use of asthma medication (controller and/or rescue medication) during the preceding 12 months. | Current neurological, temporomandibular, cognitive, or other problems, which could interfere with lung function testing | Parents judged application as 5/5 for user friendly and for assessment of whistle. Score 4/5 (median) for functionality and overall assessment. Median score was 18/20 (15-20). | N/A | Good | **Good.** The overall median (quartiles) Paediatric Asthma Quality of Life Questionnaire (PAQLQ) score was 6.4 (6.1–6.7). The children had median (quartiles) Childhood Asthma Control Test (C-ACT) total score after 4 weeks of 24 points (15.5–27) and were assessed to have a well-controlled asthma. Of 21 children, the median (quartiles) days with at least one measurement during the 4-week period were 27 (21–29.5). Median days with recordings both morning and evening were 18 (9–24), with 22 (15.5–26) days with recordings in the morning and 24 (17.5–28) days with recordings in the evening. | N/A | Feasibility study using a smartphone application to electronically rate asthma condition, and measure PEF (using mathematical algorithms). Most children had at least one daily successful recording, but not all children managed to measure PEF twice daily. Median (quartiles) days with at least one measurement during the 4-week period were 27 (21–29.5). Median days with recordings both morning and evening were 18 (9–24). The total parental score of the application was high, indicating that most parents were very satisfied with the device and the application. Parents judged application as 5/5 for user friendly and for assessment of whistle. Score 4/5 (median) for functionality and overall assessment. Median score was 18/20 (15-20). |
|  | Bui et al, 2020^25^ | 20: Cohort study | Smartphone app: *Prototype* | Attending UCLA Pediatric Asthma Center of Excellence for monitoring over a 1-week period during May –December 2018. | None described | Of the 19 completed exit surveys, most children reported being satisfied (12, 63%) or very satisfied (6, 32%) with their overall experience using BREATHE and found it somewhat easy (10, 53%) or very easy (9, 47%) to use. Parents/caretakers were similarly very satisfied (9, 47%) or satisfied (8, 42%) with their child’s overall experience. Only 2 (11%) reported being dissatisfied. Most parents agreed (13, 68%) or strongly agreed (5, 26%) that their child’s experience using BREATHE was enjoyable. Similar view were held on whether it was easy to remember using as instructed (12 [63%] agree, 4 [21%] strongly agree, and 3 [15%] disagree). When asked if using BREATHE every day was burdensome or took too much time or effort for their child, most parents disagreed (7, 37%) or strongly disagreed (4, 21%), although 7 (37%) agreed and 1 (5%) strongly agreed. Most parents (14, 74%) also did not think that carrying the kit every day was uncomfortable for their child. With respect to the smartphone app specifically, of 17 respondents, 14 (82%) and 3 (18%) thought the smartphone app was very easy or somewhat easy to use, respectively. When asked what they would like to see to make BREATHE more useful to learn about their child’s asthma and triggers, the majority of parents mentioned seeing more information and reminders. | N/A | Good | N/A | N/A | Cohort study contextualizing an individual’s symptoms and daily activities over time. BREATHE enables multi-sensor mHealth studies, capturing new types of information alongside an evolving understanding of personal exposomes. High satisfaction scores from both young people and parents. |
|  | Lin et al, 2020^26^ | 55: Quasi-experimental study | Videocall or videoconference: *Pilot* | 1. 10-17 years old with physician-diagnosed asthma  2. Evidence of uncontrolled asthma in the previous 12 months  3. From three inner-city, economically disadvantaged schools between February 2016 and November 2017 | 1. Active chronic disease other than asthma/allergic disease 2. Difficulties in contacting legal guardians for consent 3. Plans to change schools. | 100% of the participants and parents reported satisfaction of telehealth visits and rated study experience as “good to excellent”. 100% of parents and 95% of subjects agreed that telehealth visits were as helpful as in‐person visits. 100% of both parents and subjects reported being satisfied with the telehealth medical visits.100% of parents and 92% of subjects would recommend the study to others. | The school principals were supportive as they were included in the design and implementation of the study and provided letters of support providing permission for the study to be conducted at their school. | Good | **Good.** There was no significant change of the mean CASI score of all participants from baseline to post intervention. Significant improvement in daytime symptoms, night-time symptoms, and exacerbations. No asthma‐related ED visits or hospitalizations - significant improvement from baseline. At enrolment, 68% of participants had a history of two or more oral steroid requiring asthma exacerbations in the preceding year. During the study period 9.5% of participants required one course of oral steroids and none required ≥2 courses. ACT scores improved following the intervention. Before the study, 81% of participants had two or more ACT scores less than 20 in the preceding year, compared with 43% during the study period. In the3 months before the study, the rate of asthma‐related school absences per person was 1.9 days; during the study period, the rate of asthma‐related school absences per person was 1.4 days. The rate of asthma‐related school absences per person during each visit month, and demonstrates a significant trend of decline over the study period | N/A | Feasibility and efficacy study of school-based care delivery model using videocall and electronic inhaler monitoring. Very strong satisfaction scores and improvements in a number of clinical and process outcomes. |
|  | Kowatsch et al, 2021^27^ | 99: Quasi-experimental study | Text/SMS/WhatsApp/Chatbot etc: *Prototype* | 1. 10- to 15-years old with asthma  2. German-speaking  3. Access to a smartphone with Google’s Android (Version 4.1 or higher) or Apple’s iOS (9.3 or higher) operating system and internet access  4. Availability of a German-speaking motivated family member of the patient with access to a smartphone with internet capability | None described | 99 patients approached: 14 had no smartphones; 3 family members had no smartphone. 48 didn't install the app. 2 installed the app but did not start the interaction with intervention and did not provide their names. 49 (49.5%) patients started to interact with the intervention.  Feedback found that 73% felt it was easy to use, enjoyable and intended to continue working with the intervention. | Health care professionals positively emphasized the perceived ease of use and the significant supporting role of family members in this intervention. Health care professionals indicated that lack of access to smartphones, especially for young patients, was a limiting factor to further increase the reach of the intervention. | Good | N/A | N/A | Quasi-experimental study using a conversational agent–delivered asthma intervention (MAX) offering hybrid (human- and conversational agent–supported) coaching. Positive evaluation regarding acceptance of technology. Most of the conversational turns (99.5%) were conducted between patients and the conversational agent rather than between patients and health care professionals, indicating scalability. However, lack of access to smartphones, especially for young patients, was a limiting factor to further increase the reach of the intervention. |
|  | Kumari et al, 2021^28^ | 247: Cohort study | Telephone: *Pilot* | All patients under 18 years who had an appointment from 2nd April 2020 to 15th May 2020 | Data of patients who had appointments but could not be contacted due to various reasons were excluded. | 78.1% of patients scored the telemedicine services 8 or more points out of 10, with 47.1% giving a rating of 10. Eight (6.6%) respondents rated below 5. A total of 95 (78.5%) respondents wanted to continue with teleconsultation rather than in-person consultation. However, only 48 (40%) of them wanted to continue it beyond the conclusion of the COVID-19 pandemic. 87% would recommend teleconsultation to others. Fifty (45%) respondents wanted to continue with audio consultation while 61 (55%) wanted video consultation next time. | 3 out of 5 residents faced "a few problems while providing consultation via telemedicine" - not detailed further. Only one resident preferred teleconsultation over in-person visit. Two residents preferred video consultation over audio consultation for all consultations, while three would prefer only for selected cases. | Good | N/A | N/A | Ambispective observational study assessing teleconsultations in patients with respiratory diagnosis, 58% of which asthma. 83.5% had prescription refill and 8.5% treatment adjustment. Some required urgent (2.7%) and non-urgent (2.1%) clinical review. 78.5% of respondents wanted to continue with teleconsultation rather than in-person consultation but only 48 (40%) wanted to continue it after the COVID-19 pandemic is over. |
|  | MacGeorge et al, 2021^29^ | 36: Cross-sectional study | Videocall or videoconference: *Demonstration* | School nurse with:  1. Current position in a school in the state of South Carolina with a school-based health clinic  2. Based in a school for at least one year. | Not working in school for one year or longer | NA | Responders were categorised as utilizers (more than one visit monthly) or non-utilizers (less than a monthly visit). Commonly cited barriers included inadequate time due to competing tasks in both utilizers (65%) and non-utilizers (74%) as well as lack of caregiver involvement in care planning (94% of utilizers and 84% of non-utilizers). There was no statistically significant difference between groups on any of these. Of those utilizing specific, relevant telehealth services, schools scored high in perceptions of organizational readiness (n=24, mean: 24.5/30), self-efficacy (n=26, mean: 3.6/5) and comfort with identifying students eligible for SBTH (n=26, mean: 3.5/5). | Fair | N/A | N/A | The authors identified time taken to use intervention and challenges engaging caregivers as key barriers to implementation of a state-wide school-based telehealth asthma program providing care to an under-resourced population. Addressing these barriers when expanding telehealth services may promote utilization of telehealth. Despite the identified barriers, school nurses feel empowered, confident and supported in using the intervention. There may an element of bias as only schools who had already implemented the intervention were included in the study. Insights from other stakeholders and organisations who had not felt comfortable adopting the intervention would have been helpful. |
|  | Makhecha et al, 2021^30^ | 23: Cohort study | Videocall or videoconference: *Prototype* | 1. Previously received at least three biologic doses with no reactions  2. Prescribed a dose which could be given at home  3. Deemed suitable by the multidisciplinary team  4. Parents accepted to take on the role of administration supervised by video calls | None described | Feasible, safe and positively perceived by children and their families. . Parents found support provided 'particularly the video call at the time of the injection' helped '[make] them feel more confident and reduced their anxiety around administering injections'. Children were reassured that they were being closely monitored, relieved that they did not have to travel to the hospital and found the home monitoring devices easy to use. Ease of technology use was not directly assessed. | N/A | Good | **Good.** FEV1, unscheduled healthcare visits and oral steroid use did not deteriorate with home administration; however, Asthma Control Test (ACT) and Paediatric Asthma Quality of Life Questionnaire (PAQLQ) improved significantly over the first 3 months of home administration | N/A | Service delivery evaluation assessing use of video link to supervise home-administration of biologics and home spirometry. 70% of patients agreed to participate. 2/75 inaccurately administered doses, without adverse event. ACT and PAQLQ improved significantly over the first 3 months of home administration, other outcomes showed no deterioration. The intervention was seen as acceptable to patients and parents. |
|  | Mayoral et al, 2021^31^ | 104: Cohort study | Smartphone App: *Pilot* | 1. 6–16 years old with a clinical diagnosis of asthma  2. Treatment with inhaled corticosteroids (alone or with long-acting beta-agonists) for more than 6 months in the previous year  3. Access to a smartphone. | None described | Mean of the System Usability Scale scores (n=85), ranging 0 (negative)-100 (positive), was 92.9 in the three age versions of the application. No differences were observed among users of different age versions (p=0.351). The median of the overall SUS score was 95.0 in parents/guardians, 95.0 in children, and 93.8 in adolescents. All higher than the pre-identified threshold of 68. | N/A | Good | N/A | N/A | Description of pilot for a mHealth application for patients with asthma and online platform for paediatricians to monitor. All usability measures far above pre-agreed thresholds. |
| **Diabetes** | Landau et al, 2012^32^ | 70: RCT | RPM without smartphone app: *Pilot* | 1. Diagnosis of type 1 diabetes  2. 11–20 years old  3. Diagnosis at least 6 months prior  4. Willingness to maintain regular follow-up every 3 months as usual | 1. Documented learning difficulties | Patient satisfaction data (scale 1-4): 3.21 for "helping understand blood sugar level"; 3.35 for "helping parents be better informed about diabetes management of child"; 3.35 for "conversation with coordinator helped deal with other aspects of diabetes". 13 patients wanted to continue with the intervention. 7 didn't. 13 didn’t know - reasons not described. Some minor technical difficulties related to data uploading at home were reported by five patients, but the study coordinator was able to resolve those difficulties by phone. 12 of the 36 participants in the intervention group did not send blood glucose levels or sent their data three times or less during the study period and were classified as non-compliant; for three of these there was no follow-up data on HbA1c levels at 6 months. During the 6 months of the trial, intervention patients (or parent) spent an average of 71 min on the phone discussing diabetes care. | The study coordinator spent a mean time of 6 min per contact with the subjects (the participant or the parent) providing feedback. The mean time of consultation with the paediatric endocrinologist was 5 min per contact with the study coordinator. | Good | **Good.** At 6 months, mean HbA1c was 8.5 ± 1.4% for the intervention group and 8.4 ± 1.1% for the control group, p = 0.54. There was 1 episode of DKA in intervention group and no hypoglycaemic episodes in either group. There was no association between the total study coordinator contact time and change in HbA1c over the course of the study. | N/A | Although usability data was very promising, it was difficult to delineate impact of coordinator and intervention. In a secondary exploratory post hoc analysis in which non-compliant patients were omitted, mean HbA1c values for the compliant intervention group declined from 8.5 ± 1.6% at baseline to 8.15 ± 1.2% at 6 months, while in the control group, mean HbA1c values increased from 8.2 ± 1.1% at baseline to 8.4 ± 1.1% at 6 months. This difference did not reach statistical significance. |
|  | Choi et al, 2013^33^ | 31: Cohort study | Mixed. RPM and email: *Pilot* | Case managers and primary care providers at Womack Army Medical Center (WAMC), Fort Bragg, North Carolina, were invited to enrol paediatric patients with type 1 diabetes | None described. | N/A | N/A | N/A | **Good.** HbA1c, when compared to baseline, was significantly lower at 3 months (9.1% vs. 11.1%, P = 0.05) and 6.5 months (9.5% vs. 11.1%, P= 0.05). | N/A | Retrospective study with remote glucose monitoring and contact between patients and team via email. HbA1c compared with baseline lower at 3 months and 6.5 months, however this was a small study. Usability of the intervention did not feature in the evaluation. |
|  | Peña et al, 2013^34^ | 13: Cohort study | RPM without smartphone app: *Pilot* | 1. Diagnosis of type 1 diabetes for at least 1 year  2. 6 - 10 years old and Tanner stage 1  3. Multiple daily insulin injection regimen and a minimum of 2 months receiving the same insulin regimen  4. Familiarity with and use of carbohydrate counting. | 1. Associated disease, except controlled coeliac and/or thyroid disease  2. High-level competitive sports  3. Continuous subcutaneous insulin infusion.  4. Patients withdrawn from study if not uploading BGL every 2 weeks, not doing minimum number of testing 5. Patients withdrawn following nonattendance at Hba1c & anthropometry meetings, or voluntary withdrawal | 84% of users believed the time required to upload the data was acceptable and all stated that they would participate again. 92% reported observing improved metabolic control but 46% believed that the assessment phase was too short. 23% reported technical problems but no further details were provided. 1 of the 15 had technical difficulties and couldn't continue. 1 user was withdrawn as they didn't upload results frequently enough. | N/A | Good | **Good.** HbA1c significantly reduced at end of 3 month phase. Increased again after 4month review free period. Other measurements examined (High/low blood glucose index and mean blood glucose) however none were significant. | N/A | Cohort study evaluating impact of 3-month telemedicine assessment. HbA1c significantly reduced at end of 3 month phase and increased again after 4month review-free period. Other measurements were examined (e.g. high/low blood glucose index and mean blood glucose) however none showed significant change. Satisfaction was high but there were frequent technical difficulties. |
|  | Damgaard et al, 2014^35^ | 31: Quasi-experimental study | Videocall or videoconference: *Pilot* | 1. Type 1 or type 2 insulin-dependent diabetes diagnosis 2. Insulin administration by injection or pump on a regularly scheduled or sliding scale basis during the school day.  3. School without a licensed nurse present every day  4. School with appropriate technology to connect to the virtual RN.  5. The school must be able to identify an unlicensed person who can partner with the virtual RN for the management of students with diabetes during the school day. | None described. | The project resulted in statistically significant improvement in parental satisfaction and school personnel satisfaction. Parents rated project 4.71/5 for meeting expectations. Almost all the schools were at capacity with network utilization, and broadband width was not available for the required clarity of the virtual consultations. Therefore, separate Internet connections were installed. The technology consultants ordered and installed identical hardware and software. | HCP felt that the intervention improved communication, evidence-based decision making and appropriate response to patient presentations. | Good | N/A | N/A | Safety and effectiveness assessment of service linking unlicensed school personnel to registered nurses with telehealth to facilitate administration of insulin and diabetes care tasks. However assessment of safety was based on subjective opinions rather than objective outcomes. There were statistically significant improvement in parental satisfaction and school personnel satisfaction. Intervention was usable and feasible once hardware and software were upgraded. |
|  | Chorianopoulou et al, 2015^36^ | 100: Case control study | RPM without smartphone app: *Pilot* | 1. Families with children with type 1 diabetes  2. Monitored in the diabetic department of the Children's Hospital Aglaia Kyriakou in Athens. | None described. | 96% of families thought their knowledge regarding the use of the telemedicine system was adequate. 82% would recommend its use to other parents. 80% believed the system adequately supported the nursing and medical care of the diabetic child. 67% of men and 14% of women believed telemedicine improves the quality of medical and nursing care. All participants whose knowledge in using a P.C is quoted as sufficient (72%) believed their training on the use of telemedicine system was adequate. Of those not using telemedicine: 72% were informed about the existence of the telemedicine system, of which 66% believes that the system can be used for improving their child's treatment, and expressed positive opinions that the telemedicine system may be useful for prevention (64%) and direct live communication (60%). Main reasons for not using telemedicine were a belief that the system does not provide access to more experienced staff (86%) or that telemedicine did not reduce the cost (92%). Other reasons for not using telemedicine were data safety (36%) and handling difficulties (24%). | N/A | Good | N/A | N/A | Case control study looking at acceptability/feasibility of telemedicine with no outcome data. 80% felt telemedicine adequate and supported medical and nursing care of child. Mixed views with differing education levels and between men and women. |
|  | Smith et al, 2016^37^ | 14: Cross-sectional study | Videocall or videoconference: *Pilot* | Caregivers of young people with diabetes seeking care at Peninsula Regional Medical Center | None described. | Caregivers were highly satisfied with the communication/ privacy, access to care and quality of services. All agreed enabled them to see a physician sooner. All were satisfied with the overall quality of telehealth visit, that it was as good as regular in-person visit and willing to have it again. | N/A | Fair | **Poor.** The benefits of telehealth program identified by authors were immediate patient access, reduced service gaps, improved quality, additional clinical support, better patient and caregiver satisfaction and improved adherence to care standards. | N/A | Caregiver satisfaction survey of quality of telemedicine consultations. Caregivers were highly satisfied with the communication/ privacy, access to care and quality of services and that it was as good as regular in-person visit and willing to have it again. No clinical outcome data, no comparison with usual care. Limited extrapolation given small sample size and survey methods. |
|  | Brown et al, 2017^38^ | 14: Case control study | Text/SMS/WhatsApp/Chatbot etc: *Pilot* | Type 1 Kidz Young People’s Steering Group at The Great North Children’s Hospital, Kerry Camara. | None described. | 86% agreed that Flo improved their confidence in managing their diabetes. 50% would have recommended it to a friend. 86% agreed they felt less worried or anxious about their diabetes. 67% felt more supported in managing their DM. 33% felt the reminder pathway helped them to self-manage their diabetes (others stated requiring a reminder was not self-managing). Some of the 50% who would not recommend Flo felt that getting regular text messages about their diabetes to be “annoying”. | N/A | Good | **Good.** Motivation pathway - increase in frequency of glucose monitoring, reduction in HbA1c, increased number of patients with glucose in range. 86% agreed that Flo motivated them to make changes that would impact on their diabetes management. 86% felt more supported in their diabetes care. 57% felt less alone. 43% said their mood about diabetes improved. | N/A | Case control study using text messaging with healthcare professional via reminder or motivation pathway. Motivation pathway: improved number of blood glucose readings taken and in the target range, and an improvement in HbA1c levels. Reminder pathway: patients felt more supported (67%), more confident managing diabetes (50%), would otherwise forget to test their blood or take their insulin (67%). 50% would recommend the system to a friend (others found text reminders annoying). 86% agreed that telehealth motivated them to make changes that would impact on their diabetes management. Small sample size. |
|  | Losiouk et al, 2017^39^ | 72: RCT | RPM without smartphone app: *Pilot* | 1. 5-9 years old  2. Attending a specialist camp  3. selected by pediatric diabetologists practicing at five different major centers in Italy. | None described. | 49.65% parents expressed a potential improvement, 50.35% said felt TM had no impact. None of the parents said that using TM could worsen the management. Parents’ ‘‘fatigue’’, which emerged through the baseline questionnaire as the worst effect of diabetes management, was considered the most improvable item through a daily usage of the TM. Mean PQL after intervention was 78.39 (higher than baseline) | 85% felt TM could improve communication. 12% neutral. 3% sceptical. Willing to pay median 200E/yr, up to 3000E/yr. Many advantages identified. | Good | N/A | N/A | Survey of perceived usefulness of telemonitoring to parents. Baseline questionnaires: 51% showed a negative tendency, mean value of perceived quality of life was 64.13 (0–100 scale). Post-study: approximately half of the parents believed in a possible improvement adopting telemonitoring. Significant improvement in quality of life score, from 64.13 to 78.39 (p-value 1⁄4 0.0001). Free-text comments highlighted an improvement in mood, and parents would be willing to pay for the service. High number of parents appreciated the telemonitoring service and were confident it could improve communication with physicians. Good acceptability data. |
|  | Gandrud et al, 2018^40^ | 117: RCT | Smartphone App: *Pilot* | 1. 8 - 17 years old  2. Performing blood glucose testing 3 times per day on an average and using an insulin pump (Medtronic, Animas, or Omnipod)  3. Diabetes diagnosis for at least 1 year  4. Baseline HbA1c from 8.0% to 10.5%  5. Access to a family smartphone | 1. Patients using multiple daily insulin injections  2. Unwilling to use a wearable monitoring device  3. Unwilling to upload data on a routine basis  4. Non-English speakers  5. Mental health diagnoses. | N/A | N/A | N/A | **Good.** Mean (SD) 6-month %HbA1c change for (IRT) vs (CC) was −0.34 (0.85) (−3.7 mmol/mol) vs −0.05 (0.74) (−0.5 mmol/mol) overall (P = .071); for ages 8 - 12: −0.15 (0.67) (1.6 mmol/mol) vs −0.02 (0.66) (0.2 mmol/mol) (P = .541); or ages 13 - 17: −0.50 (0.95) (−5.5 mmol/mol) vs −0.06 (0.80) (−0.7 mmol/mol) (P = .056).  Three months after intervention cessation, %HbA1c changed minimally among treated children aged 8 to 12 but increased by 0.22 (0.89) (2.4 mmol/ml) among those aged 13 to 17. The correlation between number of uploads and change in HbA1c was not statistically significant. Intervention improved patient-reported diabetes-related QoL - 6.5 and 1.3 points for IRT and CC, respectively (P = .062). | N/A | RCT comparing intensive remote therapy (IRT) with conventional care (CC). Mean (SD) 6-month %HbA1c change for IRT vs CC −0.34 vs −0.05, not statistically significant. Adolescents experienced a stronger treatment effect, but had difficulty in sustaining improved control after intervention cessation- minimal change among t8-12 year olds, but increased by 0.22 in 13 to 17 year olds. Diabetes- related QoL increased by 6.5 and 1.3 points for IRT and CC, respectively (P = .062). No data on acceptability/feasibility |
|  | Guttmann-Bauman et al, 2018^41^ | 27: Cohort study | Videocall or videoconference: *Pilot* | Paediatric patients with a diagnosis of diabetes for >1 year | None described. | Details of questions not provided. The total for satisfaction scores averaged 24.5 out of 25 (n = 25). 89% of patients (23/26) found telehealth visits to be equivalent to in person visits and only 8% (2/25) expressed preference for in-person visits. | N/A | Good | **Good.** No improvement in HbA1c, saw too few ED visits to evaluate. Clinic visits increased from 1.5 to 2.6 per year, with 1.9 telehealth visits per patient. | N/A | Cohort study of new clinical service to review patients F2F once a year with videoconferencing for other visits. Some data to support the feasibility/acceptance of videoconferencing (satisfaction rated high, 89% of patients (23/26) found telehealth equivalent to in-person visits), no effect on glycaemic control, some effect (increase) on clinic attendance, small sample size, non-randomised, small period of follow up |
|  | Burckhardt et al, 2019^42^ | 20: RCT | RPM (without smartphone app) : *Pilot* | 1. Parents of children diagnosed with Type 1 diabetes for > 1 year  2. Child 2 to 12 years old  3. Sensor naïve. | None described. | RPM provided greater peace of mind. Mixed views of degree of anxiety (due to additional information). Improved sleep. Improved relationships. Improved social engagement of young people. | Continuous Glucose Monitoring at home only- no detail of interaction with healthcare professionals | Good | **Poor.** Positive impact of physical exercise. | N/A | Qualitative summary of acceptability in second phase of a randomized cross-over study using standard insulin therapy with or without CGM and remote monitoring. Remote monitoring provided greater peace of mind and parents reported improved sleep, relationships and social engagement of young people. Mixed views of degree of anxiety (due to additional information). |
|  | Döğer et al, 2019^43^ | 82: Cross-sectional study | Mixed. Phone and WhatsApp. Counselling was conducted via communication networks including the internet and smart phones: *Pilot* | Paediatric patients with type 1 diabetes treated at the Gazi University Faculty of Medicine Pediatric Endocrinology Department, Ankara | None described. | No detailed assessment. Noted in terms of frequency of options (WhatsApp/SMS/telephone), there was a preference for using WhatsApp (57.3%). Frequency of use of WhatsApp (28.0%) was higher than the other means of communication. | N/A | N/A | **Fair.** It was found that HbA1c values at baseline compared with after six months were lower in patients calling frequently (p<0.001). | N/A | Presentation of outcomes for telehealth service 'range of technologies' service. WhatsApp communication preferred by families, facilitating instant messaging (57.3%), contact with diabetes education nurse (32.9%) and consultation about insulin doses and blood glucose regulation (42.7%). HbA1c values significantly lower after six months in those calling frequently (p<0.001), compared with HbA1c values at the beginning of the study. No defined protocol of use, more observational rather than interventional study. |
|  | Kaushal et al, 2019^44^ | 20: Quasi-experimental study | Text/SMS/WhatsApp/Chatbot etc: *Pilot* | 1. Diagnosis of type 1 diabetes  2. 10-17 years old  3. Duration of diabetes greater than 6 months  4. Completed an Advanced Home Management (AHM) class within the past 6 months  5. Mobile phone with an unlimited text messaging plan. | None described. | 55% (11/20) of subjects completed the day 30 satisfaction survey. All enjoyed receiving text messages when the information was helpful, and if the frequency of the text messages was appropriate. However, pilot missed the average point accumulation target (60%). | N/A | Good | N/A | N/A | Non-randomized prospective pilot trial testing feasibility and user interface of text message system supporting newly diagnosed diabetes. Only 55% responded to survey, below pilot target. Of these, all created profiles and 86% responded to at least 2 messages per week. However small sample size with poor feedback completion and likely sample bias. |
|  | Crossen et al, 2020^45^ | 57: Quasi-experimental study | Videocall or videoconference: *Pilot* | 1. 1–17 years old  2. Diagnosis of type 1 diabetes  3. HbA1c ≥8%  4. access to the internet via a device with video and audio capability  5. Ability to connect the patient’s home glucose meter (and insulin pump/ CGM if applicable) to an internet-capable device via Bluetooth or physical cable. | English language interpreter needed | Not formally assessed. 94% “very satisfied” and 6% “somewhat satisfied” with the video visit, but not specifically questioned on the technology/comparison with usual consultation modality. 21 of 57 enrolled patients dropped out of the study: 13 before completing any video visits. Voluntary withdrawal (43%) due to insufficient time, inability of the research team to contact the patient or family (38%), and technological barriers (14%) such as loss of internet access. Difficulty with remote diabetes data sharing was reported at 20% of initial video visits and 13% of subsequent visits, and difficulty with the video application was reported at 18% of initial visits but only 2% of subsequent visits. | N/A | Good | **Good.** The cohort who completed six months experienced an increase in frequency of in-person visits, from 3.2 clinic visits/year on average at baseline to 3.7 clinic visits/year on average during the study (p = 0.04). Analysis of HbA1c values revealed that among patients completing the 6-month study period, 27 (75%) experienced a decline in HbA1c. The mean HbA1c improvement for all patients who completed the study was 0.8% (95% CI 0.2 – 1.4%). Among those who demonstrated a decline in HbA1c, the mean improvement was 1.5% (95% CI 1.0 – 2.0%). | **Poor.** Visits to clinic were estimated to require a median of 240 minutes (including transportation time), with a range of 120 to 4320 minutes – the latter for a patient who travels from a great distance and stays locally overnight to attend clinic visits. Video visits were estimated to take 30 minutes with a range of 10 to 90 minutes, and uploading diabetes devices was estimated at a median time of 5 minutes with a range of 0 to 60 minutes (Table 3). The median combined time per patient for a video visit and to upload diabetes devices was 33 minutes (range 12 – 110 minutes), and median estimated time savings for a video visit (including visit time and upload time) compared to a clinic visit was 186 minutes (range 70 – 4274 minutes). | Prospective study comparing 4-, 6- and 8-weekly video consultations. Patients completing six months averaged 4.0 video visits (SD 1.1), frequency of in-person care increased from 3.2 to 3.7 clinic visits/year (P 1⁄4 0.04). Mean HbA1c reduction at six months was 0.8% (95% CI 0.2–1.4%); 94% of these patients were “very satisfied”, 6% were “somewhat satisfied” with the experience. Difficulty with remote diabetes data sharing was reported at 20% of initial video visits and 13% of subsequent visits. Difficulty with the video application was reported at 18% of initial visits but only 2% of subsequent visits. Cost-effectiveness mentioned, but unable to make any conclusions from this. |
|  | Lim et al, 2020^46^ | 35: Cross-sectional study | Telephone: *Pilot* | 1. >13 years old  2. Enrolled on transition programme (in place pre-pandemic)  3. Diagnosis of type 1 or type 2 diabetes | None described. | 63% very satisfied with the administrative processes of telehealth service and will choose APN-led telehealth service. 70% very satisfied with duration of time spent on APN-led assessment and consultation being at ease to speak, have adequate opportunity to ask questions, and being understood over the telehealth session. Adolescents and parents reported telehealth was similar (80%) to in-person clinic, and 20% reported superior to the in-person clinics. 65% of the adolescents and 67% of parents would continue to use telehealth. Feedback suggested that spending less time in the hospital greatly reduced their anxiety around contracting COVID-19 | N/A | Fair | N/A | N/A | Limited presentation of user experience survey of telemedicine. 63% satisfied with telehealth experience and would choose this again. 80% patients and parents reported similar experience to in-person clinic, 20% reported it superior. |
|  | Odeh et al, 2020^47^ | 235: Cross-sectional study | Mixed. Telephone and whatsapp: *Pilot* | 1. Diagnosis of type 1 diabetes  2. 1–21 years old  3. Attending the outpatient clinic at Jordan University Hospital | None described. | Not assessed in detail- survey on caring for a child with T1DM during Covid. Of respondents, 39.6% needed to contact medical team during 10 week lockdown, of which 85.5% of parents described the accessibility to members of the medical team through phone calls and/or WhatsApp as smooth. | N/A | Fair | N/A | N/A | Tangential data on telemedicine as part of a wider discussion of pandemic response. The authors write that "the easy accessibility to the medical team through telemedicine and the ongoing management ... enabled timely medical advice and prevented further acute metabolic complications that otherwise could have occurred". The vast majority (85%) of families described the experience as positive. |
|  | Predieri et al, 2020^48^ | 62: Cohort study | Mixed. RPM, video and telephone: *Pilot* | 1. ≥1 year old at recruitment  2. School or pre-school attendance 3. Type 1 diabetes diagnosed in clinic  4. Regular 3-months clinical follow up attendance  5. Regular self-monitoring of glucose with specified device  6.Sensor use ≥70% over the most recent 14 days  7. No transition in insulin regime during study periods. | 1. Other types of diabetes  2. T1D onset and diagnosis in another centre  3. Other types of sensor  4. Patients using Dexcom G6® receiver instead of mobile phones  5. Documented chronic complications. | N/A | N/A | N/A | **Good.** Median value of the glucose management indicator decreased but statistically insignificant. Glucose standard deviation (p<0.0001) and coefficient of variation (p=0.001) improved across the study. Median time in range increased from 60.5% to 63.5% (p=0.008), time above range decreased from 37.3% to 34.1% (p=0.048), and time below range decreased from 1.85% to 1.45% (p=0.001). | N/A | Retrospective and longitudinal observational study during covid pandemic, with remote glucose monitoring and video calls. Improved glycemic control during lockdown, suggests that 'real-time CGM, the continuous parental management, and the telemedicine can display beneficial effects on T1D care' despite patients being confined to their homes and limited to exercise. Predominantly observational paper rather than study assessing intervention. No acceptability/ feasibility data. |
|  | von Sengbusch et al, 2020^49^ | 240: RCT (multicentre) | Videocall or videoconfereence: *Pilot* | 1. Diagnosis of type 1 diabetes for 6 months or longer  2. 1-16 years of age  3. CGM system with multiple daily injections (MDI) or insulin pump therapy(CSII)  4. Access to the internet and a computer  5. Sufficient language skills in German, English or Turkish. | 1. Private health insurance  2. Insufficient language skills  3. Unclear housing situation  4. Health insurance didn't sign contract  5. No CGM system  6. Received telemedicine before this intervention. | Not formally assessed. 17% discontinued telemedicine prematurely, no specific reason given in most cases, frustration with technical problems led to early termination in some. | N/A | N/A | **Excellent.** After adjustment for covariates, the decline in HbA1c was 0.11% lower in the Intervention Group (IG) than that in the Waitlist control Group (WG), (95CI −0.30 to 0.09%). IG group had better treatment satisfaction improvement, and better decrease in diabetes burden for main caregivers | N/A | 6-month multicentre trial. Participants quasi-randomized to intervention (IG) (monthly video consultation) or waitlist-control group (WG). No significant HbA1c difference after 6 months, but decreased diabetes burden and increased treatment satisfaction for the parents. After 12 and 15 months, a significant HbA1c improvement was found. High quality study but with no feasibility/acceptability data. |
|  | Bassi et al, 2022^50^ | 290: Cross-sectional study | Videocall or videoconference: *Pilot* | 1. Type 1 diabetes according to ADA criteria | 1. Patients/ caregivers unable to understand, read or write in Italian | 90% felt able to explain medical problems well enough via televisit, statistically significant difference between patients (93.5%) and care-givers (83.6%), 74% felt absence of physical contact was not a relevant problem, and 92% were satisfied with overall quality of service provided by televisit. 68% thought televisits were an adequate modality of assistance for their disease, and 80% were willing to continue some follow-up visits via videocall, keeping appointments in person at longer intervals. 77% of parents felt televisits granted them savings in time/money and/or time off work. | N/A | Fair | **N/A** | N/A | Aim to evaluate satisfaction in using videocalls for patients with Type 1 diabetes. Majority found to be acceptable and would continue use with longer intervals between face-to-face appointments. Ability to convey medical details of patients reported better by patients than caregivers. |
|  | Frieltz et al, 2022^51^ | 240. Quasi- randomized multicentre study | Videocall or videoconference: *Pilot* | 1. Use of a CGM system |  | N/A | N/A | N/A | **Fair.** Video consultation as an add-on service resulted in a small but nonsignificant reduction in the overall costs | **Fair** large control group of children (AG) equipped with CGM to serve as a baseline. N AG’s data were deducted at a time when inaccuracies in the data collection of CGM systems existed in the participating health insurances, such that the AG’s data were only reliable for medication comparisons but not accurate enough for the comparison with medical supplies. | However, the evaluation of the virtual clinic cost data showed a small shift in costs away from hospital treatment and a large increase in costs of medical devices (IG € 2,244; WG € 2,599; AG € 1,262). Price reductions in the area of rtCGM with an increasing number of providers as well as extra outpatient services (video consultation) may change the shares of the cost categories again in the next few years. |
|  | Troncone et al, 2022^52^ | 397. Cross- sectional study | Videocall or videoconference: *Pilot* | 1. Type 1 diabetes 2. 10-18 years 3. With a primary caregiver literate and capable of filling out online questionnaires | 1. Other illnesses (severe disability due to disease, significant comorbidity, other diagnosed diseases). | No differences between video consultation and in-person participants regarding care satisfaction dimensions (doctor's availability, interpersonal skills, technical skills, information provision (p>0.05), nor in doctor-patient relationship for tasks (p=0.506) and bond (p=0.828). Parents evaluated patient-doctor agreement on explicit goals of treatment higher in video consultations than in person (p=0.009, d=0.211). No differences found between video consultations and in-person visits in physician empathy perceived by patients (p=0.096). | N/A | Fair | N/A | N/A | Evaluation of doctor-patient relationship as perceived in video consultations vs. in -person visits for patients with type 1 diabetes, focussing on caregivers' perceptions. No differences between video consultations and in-person visits in terms of care satisfaction, doctor-patient relationship in agreement on tasks and bond, as perceived by parents, and physician empathy as perceived by patients. |
| **Epilepsy** | Bahrani et al, 2017^53^ | 465: RCT | Telephone: *Demonstration* | 1. Stable epilepsy patients older than 10 years of age 2. With telephone access  3. In centre's care for at least 6 months  4. Treatment plan had been finalized. | Serious co-morbidities | Satisfaction >90% in telephone group. Questionnaire didn't ask for patient's preference on telephone call vs face to face appointment, or if more/equally/less satisfied with the modality randomized to. | N/A | Good | **Good.** No difference in breakthrough seizures | **Good.** INR 865 more in face-to-face group | Randomized parallel group study comparing telephonic (TC) with conventional clinic review. No significant difference in breakthrough seizures. Mean consultation time 10 min in TC group, 22 hours in face-to-face group. Clinic on average cost INR 865 more than TC. >90% satisfaction in TC group. Significantly more lost to follow up in conventional clinic. |
|  | Conde-Blanco et al, 2020^54^ | 66: Cross-sectional study | Videocall or videoconference: *Pilot* | Online survey sent to the members of the Spanish Epilepsy Society and the members of the Epilepsy Study Group of the Catalan Neurological Society. | None described. | Patients/families were not directly asked. Respondents: 80.3% adult neurologists, 19.7% pediatric neurologists. The view of doctors on patients' impressions was sought: 78.8% of doctors stated that some patients would like to have this type of telephone/videoconference follow-up in the future. Only 10% felt that very few patients would be interested in this system. | 84.8% would be open to telematic visits in the future. 42.4% performed first visits also with telemedicine, 38% preferred to postpone them to see the patients onsite after the lockdown period. Regarding sudden unexpected death in epilepsy (SUDEP), the great majority (84.4%) reported that they preferred the discussion to be held with the patient and family during a normal visit. | Fair | **Fair.** 42% reported less frequent medication change compared with onsite visits, while 24% reported similar frequency. 37% reported shorter time than onsite visits, 24% reported similar, 5% reported longer. | N/A | Survey sent to members of Spanish epilepsy societies, surveying adult and paediatric neurologists on telemedicine during Covid-19 pandemic. 88% used telephone calls for clinics, 4.5% videoconference. Medication changes less frequent than with in-person visits for 66%. 84.8% open to telemedicine use in the future. Concern regarding sensitive conversations not being in-person. No patient data. |
|  | Dozières-Puyravel et al, 2020^55^ | 145: Quasi-experimental study | Videocall or videoconference Paediatric attendees at rare epilepy center. Variety of diagnoses: *Pilot* | 1. Scheduled clinic in the rare epilepsy center  2. Diagnosis with an Orpha code. | 1. First clinic  2. EEG required  3. Physician preference | Parents felt TC better than a clinic pinpointing the gain of time and the absence of travel. 44.6% felt that TC could replace in-person clinics. 12% felt that the physician is more distant with TC. Preference for next appointment: 49.5% wanted video consultation; 41.4% wanted at-hospital clinic; 9.1% telephone consultation. | Physicians confident for TC at next visit for 74.8% of patients. Of 151 TCs, physicians would like to be able to perform physical examination for 47, measure the weight for 62, evaluate psychomotor development for 43, and evaluate the behavior for 29 children. | Good | N/A | N/A | One month prospective survey of telephone consultations (TC). Physicians confident for TC in next visit for 74.8%. Limitations: absence of physical examination, weight, and psychomotor development evaluation. Parents felt TC better for gain of time and the absence of travel, however half would prefer clinic for next appointment. |
|  | Panda et al, 2020^56^ | 153: Cohort study | Mixed. Whatsapp text and video: *Pilot* | 1. Children (1month-18 years) with epilepsy, with query unrelated to epilepsy.  2. Children with any primary neurological disorder, with query related to epilepsy.  3. Teleconsultation through phonecall/SMS/WhatsApp. | Telephone call was disconnected before teleconsultation complete and could not be connected afterward | 147 (96 %) caregivers were satisfied with medical advice provided over the telephone. Acceptability of technology to families not explored. 5 calls interrupted due to technical glitches. Of 58 participants belonging to lower socioeconomic status, 19 were requested to supplement with picture or recordings- 12 able to comply, 7 did not have suitable device and the treatment was prescribed based on the best possible clinical judgment of the investigator. | N/A | Good | N/A | N/A | Prospective follow-up study using telephone calls and texts in line with India's new guidelines implemented during covid.96% caregivers satisfied with advice, but acceptability of technology not explored. Not all participants had appropriate technology (e.g. to send photographs). Considered feasibility and significant clinical events reported during study period, but no comparison with usual care. |
|  | Nieman et al, 2020^57^ | 350: Cross-sectional study | RPM (without smartphone app): *Demonstration* | Young people with epilepsy | None described. | 139 telemedicine visits provided but results only n=57. 98% saved time relative to clinical visit. 89% No missed school or work. 98% would choose telemedicine again. 75% rate travel to epilepsy centre as expensive. 91% questions and concerns addressed well through telemedicine. No presentation of number of patients not wanting to use this platform/if any had tried but been unsuccessful with internet connection etc. | N/A | Fair | **Fair.** No-show rate for visits with epilepsy specialists was halved (15% to 7%). | **Fair.** 98% saved time relative to clinical visit. 89% No missed school or work. 98% would choose telemedicine again. 75% rate travel to epilepsy centre as expensive. | Post-telemedicine survey. Paper's focus is describing the virtual platform and use, in which a survey of 57 reports majority saved time, didn't miss school or work, and would choose telemedicine again. However, this is not focus of the paper and methodology for selecting patients and survey is not presented. |
|  | Trivisano et al, 2020^58^ | 3321: Cross-sectional study | Mixed. Telephone, email, text, video: *Pilot* | Children diagnosed with “epilepsy” in last 5 years at the Bambino Gesù Children’s Hospital in Rome. | None described | 18.9% had a face-to-face visit, 25.1% had a structured remote-consultation of which 93.9% were satisfied, driven by convenience of consultation at home (50.0%) and a shorter waiting list (23.9%). 6% unsatisfied with remote consultation: problems with internet connection (61.1%) and inadequate for their purposes (20.2%). Perceived advantages included avoidance of long journeys and economics. 48.3% considered remote consultation appropriate to discuss locally performed tests and planning of examinations (70.9%), and delivery of reports (79.7%). | 93.9% were satisfied. 67.2% considered continued remote consultations advantageous. Remote consultations were considered inappropriate for neurological assessment (60.7%) and psychological consultation (59.6%). | Fair | **Fair.** 66.4% reported unchanged seizure frequency. 13.3% increased, 20.3% decreased. 87.5% had no issues getting drugs. | N/A | This study reports a questionnaire looking at the overall impact of Covid-19 pandemic on paediatric patients with epilepsy, including experiences of remote consultations, but this is not the focus of the paper. No detail provided of which patients had virtual consultations, methods or purpose of virtual consultations, and decision making for which patients had appointments. Respondents not asked preference. |
|  | Anuszkiewicz et al, 2022^59^ | 402. Cross- sectional study | Telephone. *Pre-prototype* | 1. Diagnosis of G.40 in ICD or diagnosis "epilepsy" according to ILAE definition 2. Attended developmental neurology outpatient clinic between March and December 2020 | 1. Unconfirmed diagnosis of epilepsy 2. Diagnosis made in 2020 (unable to compare before and after pandemic outbreak). 3. Only one provoked episode of seizure in the past attributed to transient states like fever, haemorrhage, and infection. | Fifty-eight percent of patients were satisfied with telemedicine. Poorer satisfaction was connected with less frequent visits, cancellation of scheduled appointments, and lack of help in case of need in an emergency situation | N/A | Fair | N/A | N/A | Epilepsy course in pediatric patients seems to be stable during COVID-19 pandemic. Sleep disturbances and changes in a child’s behavior may be related to increase in seizure frequency. Telemedicine was reported an effective tool for supervising children with epilepsy but there was a very high dissatisfaction rate. Patients should be informed about possible ways of getting help in urgent cases |
|  | Armeno et al, 2022^60^ | 37: Observational study | Videocall or videoconference: prototype | 1. 0-18 years 2. Drug-resistant epilepsy 3. Started classic ketogenic diet between January 2020 and April 2021 | 1. Children in whom the diet was initiated during status epilepticus or inpatient basis were excluded | 10 families sent the survey back (55.5% response rate). All families (10/10) thought the on-line consultations were helpful when initiating KDT and felt supported by the team during the treatment and nine responded that the modality was effective. All patients indicated that the educational materials (PowerPoint presentations) were useful and 5/10 thought educational videos would have been helpful. All 10 families felt positive about the online training to avoid going to the hospital, although 3/10 also stated that the training period was stressful.  Authors acknowledge selection bias was probably present as patients selected on ease of access to hospital. | N/A | Fair | **Fair.** No significant differences in ASMs or seizure control were found between the two groups at 3 (p 0.115) and 6 months (p 0.382) | **N/A** | modality was as effective as outpatient management in terms of seizure management. Positive aspects of online initiation were the absence of travel time, a reduced waiting list, more efficient appointments and an individual approach, and good coordination of the visits by zoom regarding medical and educational meetings. Nevertheless, there were technological limitations and inadequate anthropological measurements and clinical information |
|  | Gali et al, 2022^61^ | 61: Quality Improvement | Videocall and telephone call: Pilot | 1. 2-21 year old 2. Epilepsy and/or seizure disorder 3. Willingness to complete a telehealth visit with their paediatric neurologist, and having previously completed at least one in-person clinic visit | 1. First-time seizure or febrile seizures | 97% patient satisfaction | N/A | Fair | N/A | **Fair**. Missed school hours was reduced with telehealth by 49% (p<0.0001) and missed work hours by 48% (p<0.0001). Statistical significance (p<0.0001) also found with miles travelled and self-reported out-of-pocket expenses: telehealth visit mileage (32 miles) compared to an in-person visit mileage (49 miles). Out of pocket expenses for telehealth were US$35 compared to US$176 for in-person visit. | Four practice partnership groups were involved, utilising telehealth (Videocalls or telephone calls) at remote sites to liaise with regional specialists. Patients travelled to a site for telehealth consultation with specialist that would usually require further travel for face-to-face appointments. |
| **Nephrology** | Trnka et al, 2015^62^ | 168: Cross-sectional study | Mixed. Video, telephone, email: *Pilot* | Paediatric nephrology telehealth consultations through the Queensland Telepaediatric Service between January 2004 to December 2013 | None described | NA | N/A | N/A | N/A | **Fair.** Estimated cost savings in 2013 of $31,837- average of $505 per consultation. | Retrospective audit of telehealth (video, telephone, email) consultations- 318 consultations occurred for 168 patients. Congenital anomalies of the kidney and urinary tract (30 %), followed by nephrotic syndrome (16 %), kidney transplant (12 %), and urinary tract infection (9 %) were the most common diagnoses. The estimated cost savings associated with telehealth were $31,837 in 2013 (average saving of $505 per consultation). |
|  | Aydemir et al, 2021^63^ | 69: Cross-sectional study | Videocall or videoconference: *Pilot* | 1. 0 - 21 years of age  2. Under the care of pediatric nephrology or other specialties. | 1. Patients requiring urgent critical care 2. Primarily admitted to obtain diagnosis. | Overall, patients reported telemedicine visits as positive or neutral and with similar levels of satisfaction, compared with in-person visits. 85% felt comfortable in the interview, and 94% found it useful. Time spent setting up 1-15mins in 68%. 81% had no technical issues- rest complained of visual and connection problems. 15% had technical difficulties. 60% of interviewees would not prefer telemedicine save for during the pandemic. | Physician satisfaction score 1-10, 65% scored 9, 22% scored 10. | Fair | N/A | N/A | Prospective study across five paediatric subspecialties. Physician satisfaction 87% and considered to be feasible. Main concerns were inability to perform physical and laboratory examinations. Patient satisfaction 99% but 15% patients had technical difficulties and 60% would not prefer telemedicine save for during the pandemic. Main benefit patients reported was no travel time. |
|  | Trace et al, 2020^64^ | 20: Cross-sectional study | Videocall or videoconference: *Pilot* | 1. Purposive, maximum-variation sample selected from the South West Paediatric Nephrology Network.  2. All families had received previous dietetic support via e-mail, telephone or in-person | None described | Most families did not report audio-visual issues. Minor time-lags occurred in six VC, not felt to have affected the experience. VC at home gave a sense of familiarity and intimacy, providing a more comfortable environment for children.  Parents commented that in a face-to-face clinic, there is no time to process information, but screen-sharing, enabled understanding of growth patterns, giving parents encouragement to continue dietary interventions.  Engagement with VC was good but influenced by the childs’ age. Younger children enjoyed the novelty of using the computer and older children enjoyed the familiarity of screen-based conversations. Preschool children were not anticipated to have direct involvement and was disengaging when attempted. VC was wanted supplement not substitute local dietetic care. All children reported a desire for future VCs.  Dietician not always aware who else was present raising confidentiality concerns. Connection problems in all but two interviews, usually lasted a few minutes. Browser updates often required to enable connection, needing telephone support from the dietician. | Dietitian interviews confirmed parents’ reports that school-age children engaged positively. | Fair | N/A | N/A | Purposive sample comparing usual care with videoconsultations. Satisfaction with videoconsultations was high, no data security fears and only minor privacy concerns. Parents praised efficiency and improved access to specialist advice, requesting that videoconsultations supplement care. Children preferred videoconsultations outright. |
|  | Pooni et al, 2021^65^ | 20: Cross-sectional study | Videocall or videoconference: *Pilot* | Patients, families, and providers participating in telemedicine pediatric subspecialty follow-up visits between March 23, 2020 and May 05, 2020 during the COVID-19 pandemic | 1. New patient referrals  2. <5 years of age  3. Non-English speaking patients/ guardians  4. Cancelled or didn't attend appointments  5. Subjects unable to connect to the video visit platform  6. Declined such visits prior to the consenting process. | 17.5% had technical issues - difficulty logging on or poor audio quality. User feedback via Makoul CAT and RAND VSQ, but without asking for preference and comparison to face-to-face consultations. Those with connectivity issues and those that “did not attend” were excluded from recruitment - therefore the study could not capture the impact of this variable. | 86.1% of providers felt they could gather appropriate information and overall rated the visit highly (mean=8.1/10).  100% of providers felt video was a safer medium given the recent pandemic. 72.7% of providers reported that they were able to complete elements of the physical exam virtually. Positive clinical aspects included observation of patient in home environment, some had technical issues (e.g., “...virtual waiting room was a little unclear). | Fair | N/A | N/A | Mixed-methods study of telemedicine visits during covid-19 pandemic across pediatric endocrinology, nephrology, orthopedic surgery, and rheumatology. Providers felt it safer for their patients to conduct visits by video; 72.7% reported completing some component of a clinical exam. Patients rated being respected by clinical staff and showing care and concern highly. Mean overall satisfaction was 86.7 ± 19.3%. 17.5% had technical difficulties. Those with connectivity issues were excluded. |
|  | Raina et al, 2021^66^ | 597: Cross-sectional study | Videocall or videoconference: *Pilot* | Patients or physicians in paediatric nephrology services from 62 centres across North America. Data were collected from March 30, 2020 to August 30, 2020 | None described | 87% overall satisfied with quality of service provided by telemedicine. Virtual visit better for: travel time (85%), finding a convenient time (71%), and time waiting for clinician (53%). No perceived difference between office and virtual visits for: time spent with clinician (85%), cost (50%), quality of visit (48%). 80% thought office visit better for showing a clinician a physical problem. 50% satisfied they didn't need assistance when using the system- further details not provided. 33% thought office visit was better for cost of the visit. | The majority of physicians expressed satisfaction with the telemedicine experience. 5% (n = 10) were disappointed with the clinical aspect of telemedicine, 4% were disappointed with telemedicine overall | Fair | N/A | N/A | Survey of telemedicine experience during covid-19 pandemic. Patients reported positive experiences: logistically easier than in-person visits and quality of visits were equivalent to in person visits. Physicians used a wide variety of online systems to conduct appointments. Most physicians' concerns related to technological issues and the ability to procure physical exams and/or laboratory results. |
|  | Qiu et al, 2022^67^ | 11: Cross-sectional study | Videocall or videoconference: *Prototype* | Pediatric nephrology patients aged 11–18 years of age and their caregiver with at least one telemedicine encounter | None | Consult specific factors ( Comfort, ease of describing symptoms, encounters more personal, long waits for appointments, staying close to home, like talking in person, more than just a phone call) Context specific factors (Travelling is a hassle, Likes a trip, Option relieves stress, Telemedicine creates convenience, telemedicine is cost efficient, telemedicine is safe) | N/A | Fair | N/A | N/A | "Consult", "context" and "other mediating factors". We determined that preference for telemedicine for both adolescent and caregiver is dependent on consult specific and context-specific factors, with adolescent diagnosis and weather conditions being the main modifying factors. For dyads less than 500 km away from the center slightly more adolescents favored in person visits than their caregivers. No difference in the preference between telemedicine and tertiary care center if the adolescents lived more than 500 km away. Indiscriminate transfer to chronic care predicated on mainly telemedicine approach is not compatible with user expressed attitudes (especially adolescents). Understanding the preferences and perceptions of telemedicine, by users, is essential for best utilization of virtual care of the adolescent in the out-patient setting |
|  | Clark et al, 2022^68^ | 175: Cross-sectional study | Videocall or conference: *Pilot* | 1. Surveys distributed to providers and caregivers at centres participating in the SCOPE collaborative 2. Children under their care receiving maintenance peritoneal dialysis | 1. Caregivers of children on haemodialysis. | 58% of caregivers felt child's needs were addressed in a manner similar to an in-person visit, however only 51% desired additional telehealth visits in the future. Of those who wanted additional telehealth visits, greatest percentage (43%) felt that the ideal frequency of PD telehealth visits was alternating monthly telehealth with in-person visits, however 42% felt that telehealth visits should not be conducted for paediatric PD patients. | Providers (64%) felt time efficiency with telehealth was better or much better compared with in-person visits, with no difference across provider discipline. 47% felt the ideal frequency was alternating monthly telehealth visits with in-person visits. Benefits: ability to observe the home, family centred, ability for all team members to meet the family together, ability to review meds with access to the medication bottles. Challenges: inadequate/lack of physical exam, inability to visit. in-person, inadequate/lack of PD catheter exit site exam. | Fair | N/A | N/A | Survey for providers and caregivers of children on home peritoneal dialysis. Provider challenges included lack of physical exam, inability to visit the patient/family in-person, and inadequate/lack of PD catheter exit site exam. Only 51% of caregivers desired future telehealth visits. Benefits of telehealth for caregivers included no travel, shorter time required for visits, easier to care for other children more comfortable for patient, and no time off from work, however only 51% desired future telehealth visits, and 42% felt telehealth visits should not be conducted for paediatric peritoneal dialysis patients. |
